# Supplementary figures and images for: Unique growth and morphology properties of Clade 5 Clostridioides difficile strains revealed by single-cell time-lapse microscopy
Source: PLoS Pathog. 2025 May 21;21(5):e1013155. doi: 10.1371/journal.ppat.1013155 (PMC12140426; doi:10.1371/journal.ppat.1013155)

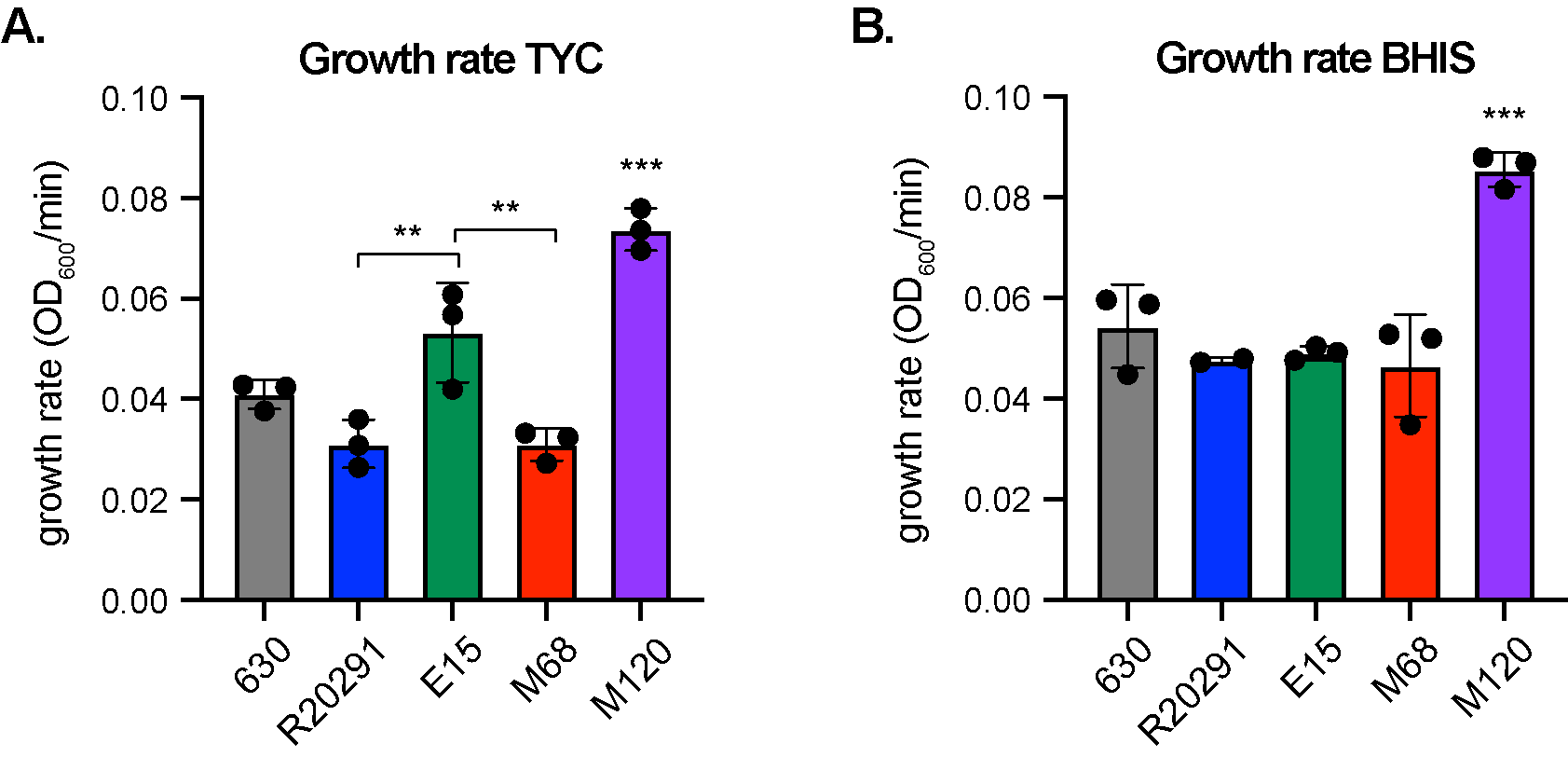

Supplement: S1 Fig — (TIF) [file ppat.1013155.s001.tif]

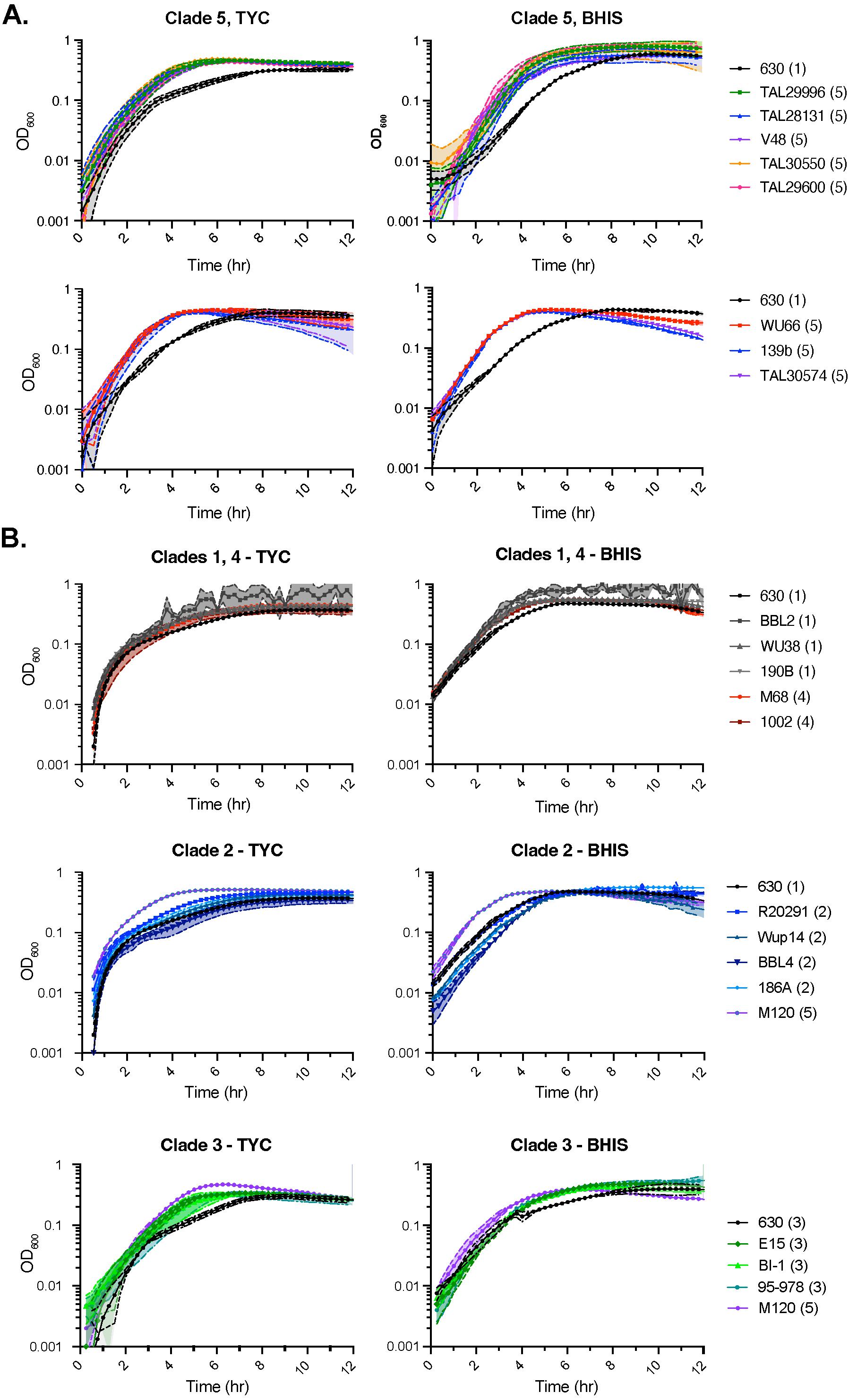

Supplement: S2 Fig — (TIF) [file ppat.1013155.s002.tif]

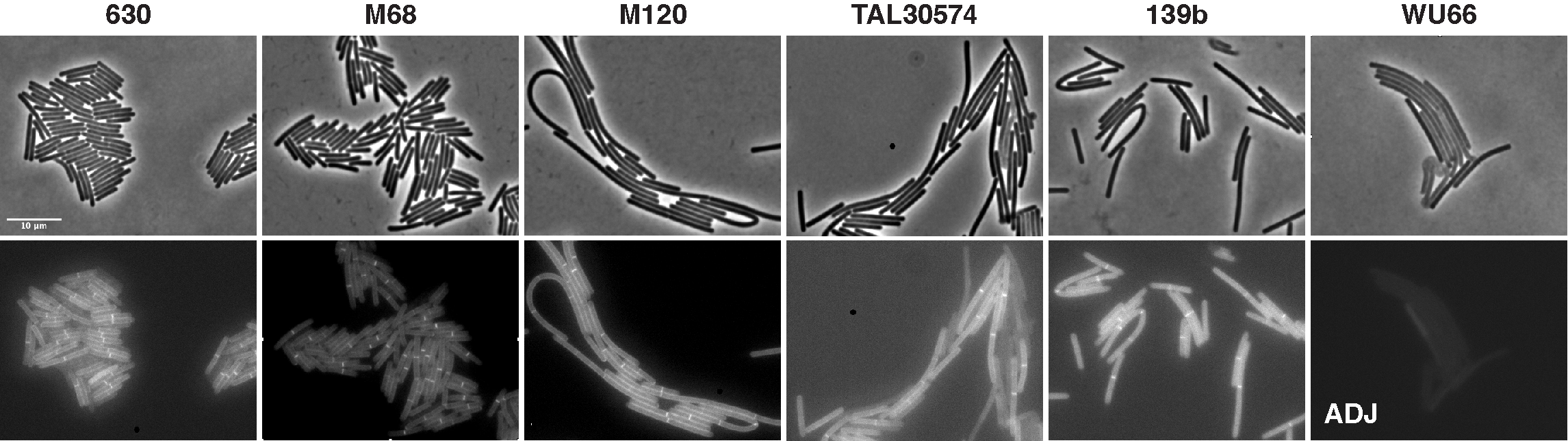

Supplement: S3 Fig — (TIF) [file ppat.1013155.s003.tif]

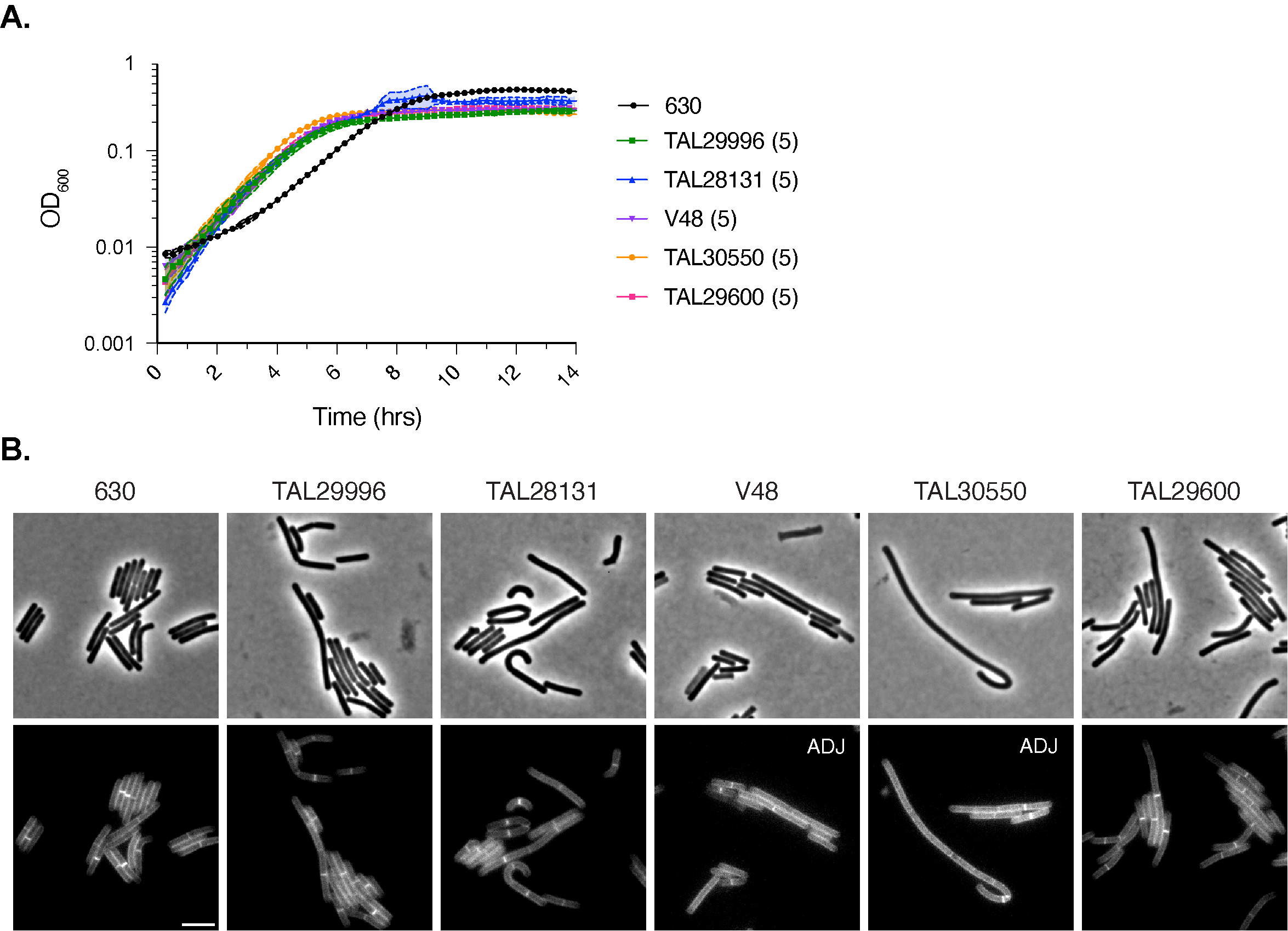

Supplement: S4 Fig — (TIF) [file ppat.1013155.s004.tif]

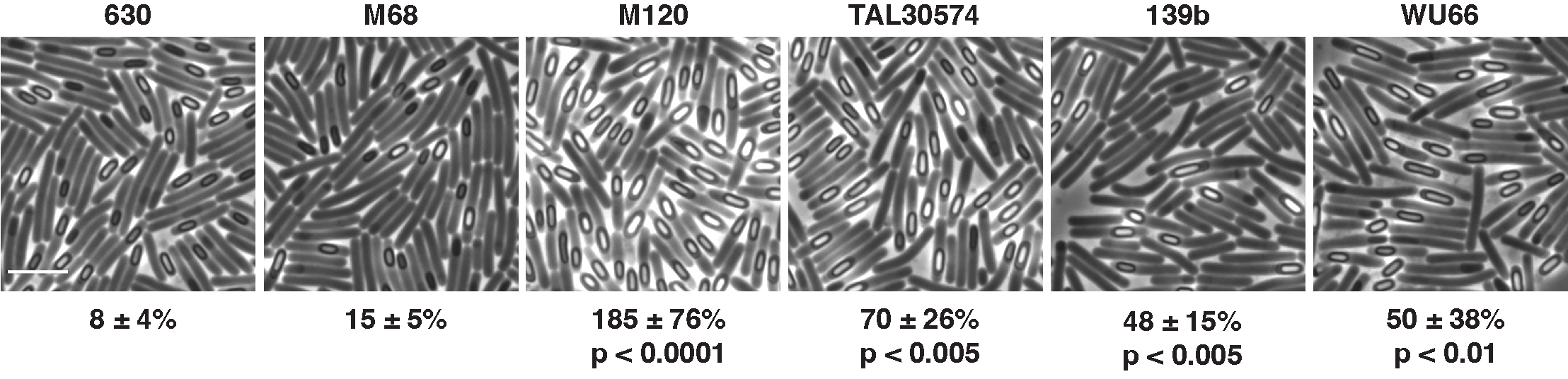

Supplement: S5 Fig — (TIF) [file ppat.1013155.s005.tif]

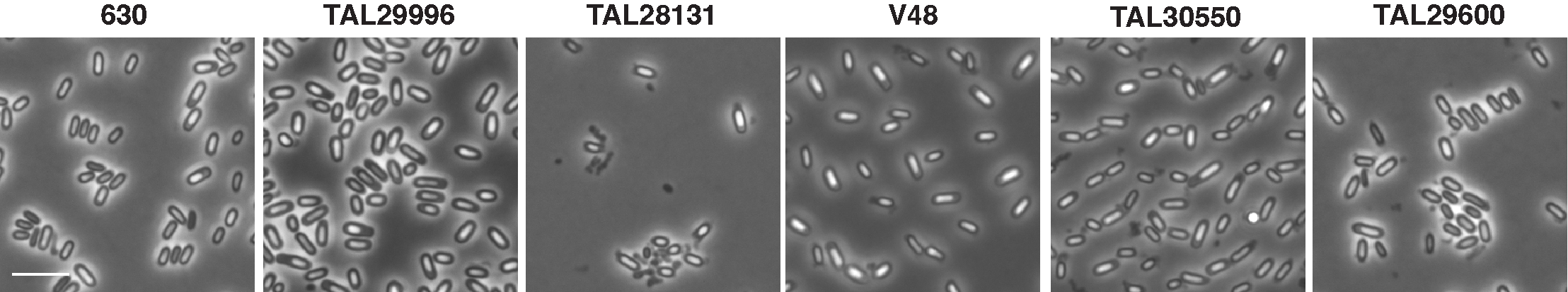

Supplement: S6 Fig — (TIF) [file ppat.1013155.s006.tif]

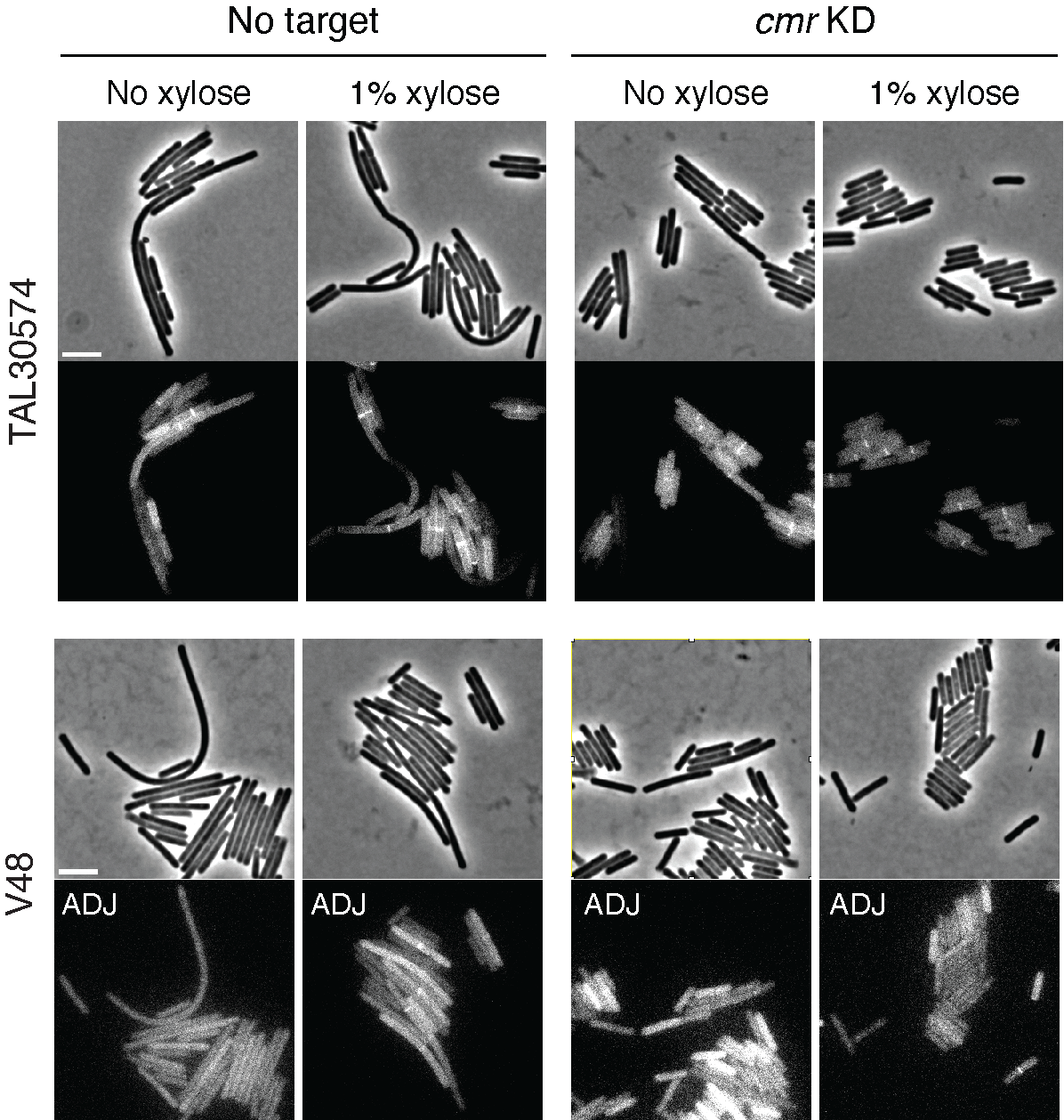

Supplement: S7 Fig — (TIF) [file ppat.1013155.s007.tif]

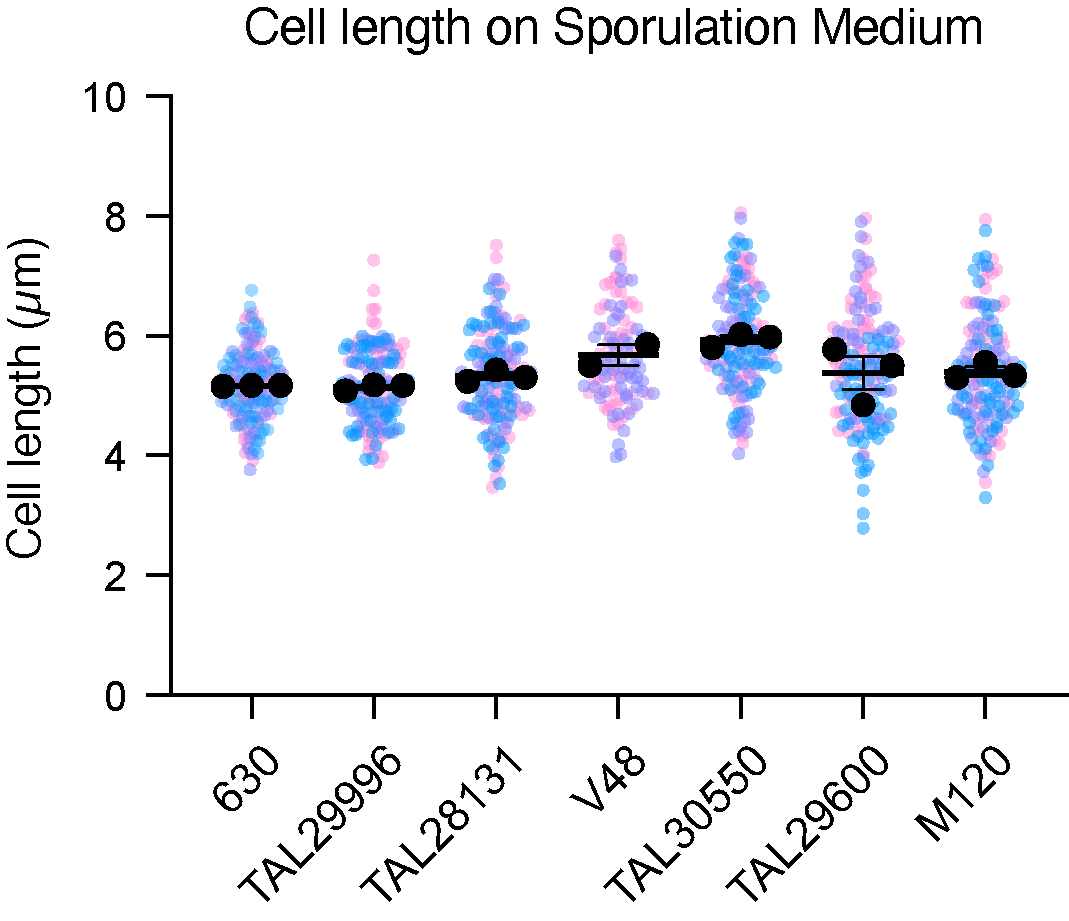

Supplement: S8 Fig — (TIF) [file ppat.1013155.s008.tif]

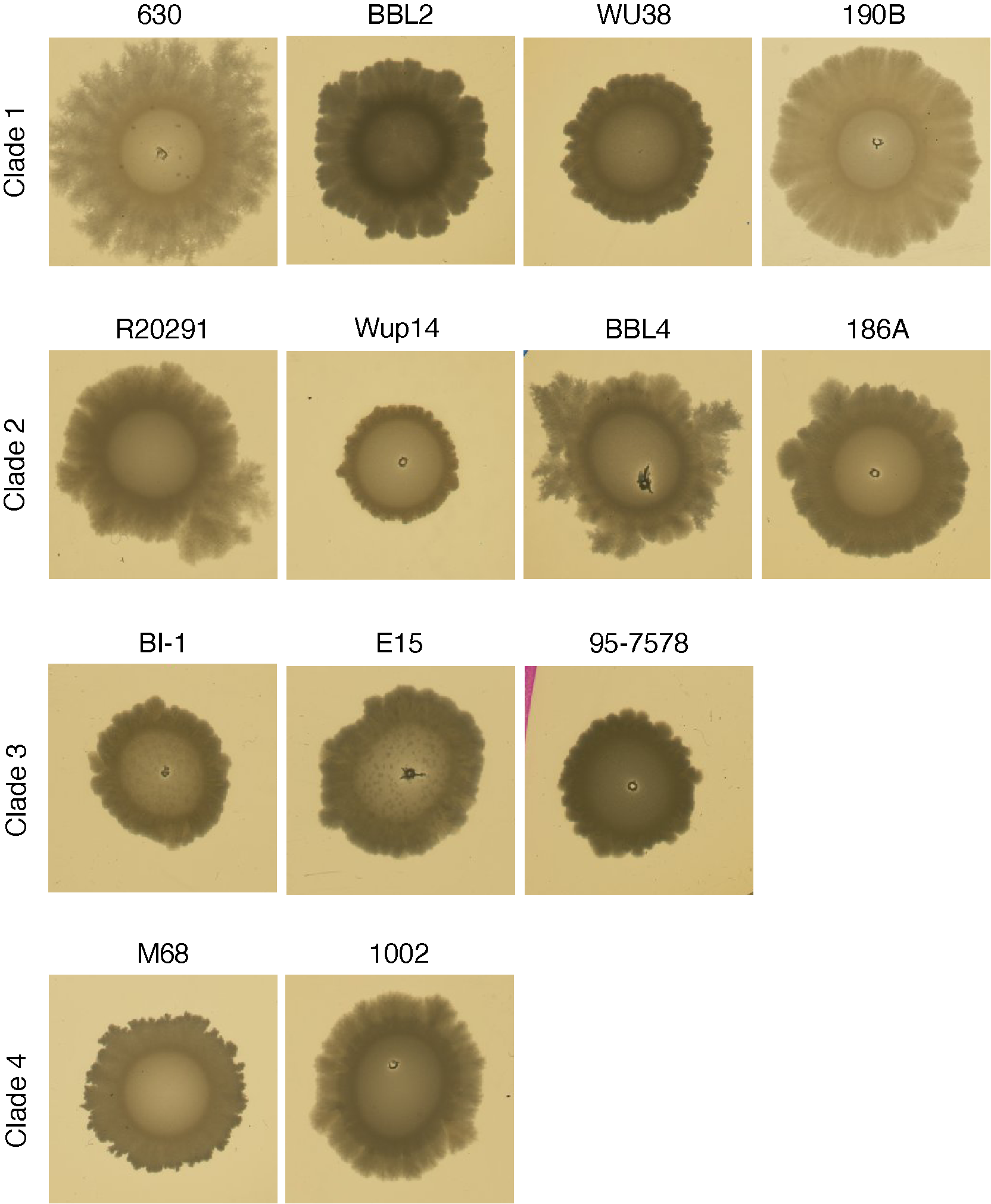

Supplement: S9 Fig — (TIF) [file ppat.1013155.s009.tif]

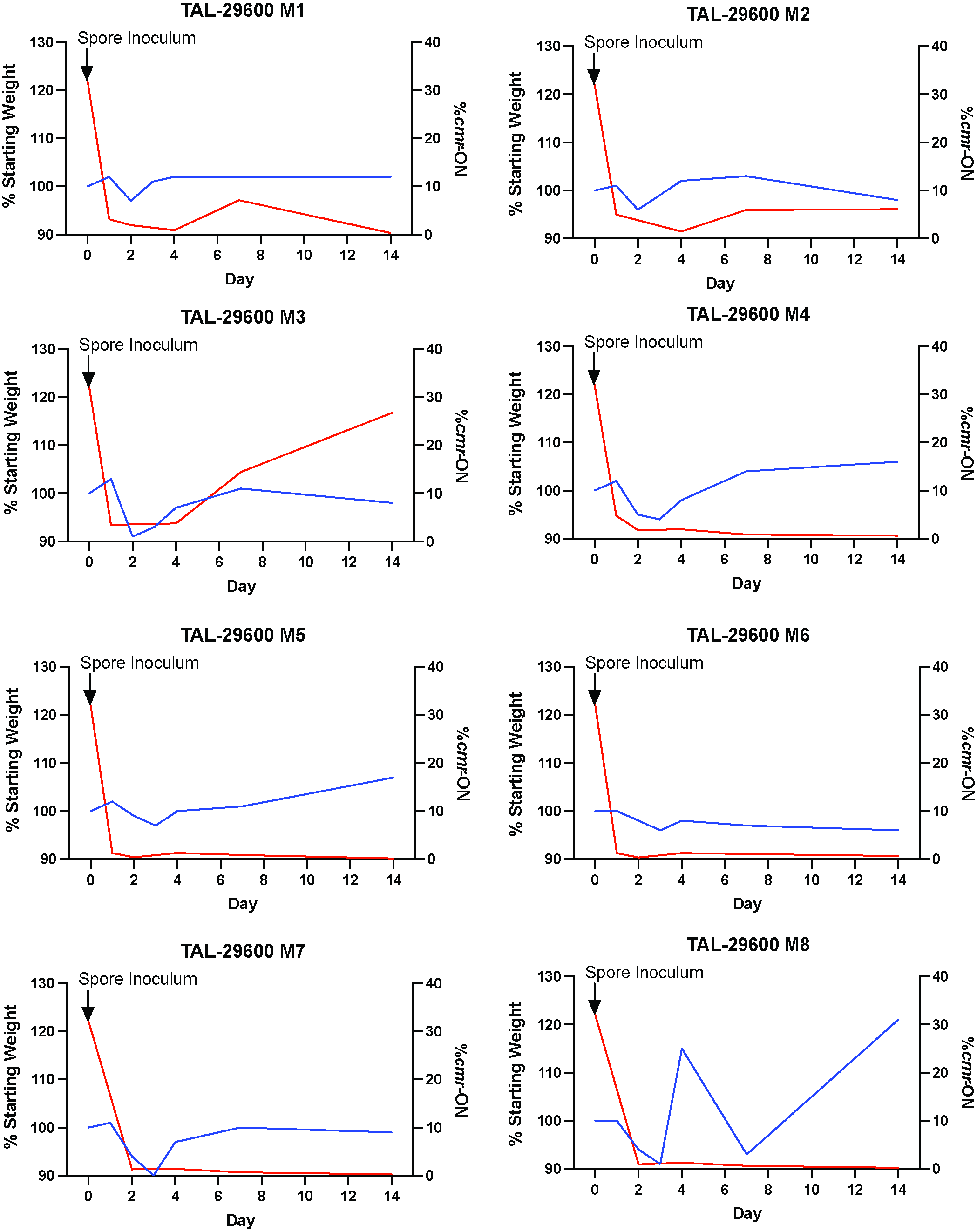

Supplement: S10 Fig — (TIF) [file ppat.1013155.s010.tif]
