# Supplementary material for: Unique growth and morphology properties of Clade 5 Clostridioides difficile strains revealed by single-cell time-lapse microscopy
Source: PLoS Pathog. 2025 May 21;21(5):e1013155. doi: 10.1371/journal.ppat.1013155 (PMC12140426; doi:10.1371/journal.ppat.1013155)
Supplement: S1 Table — (PDF) [file ppat.1013155.s011.pdf]

S1 Table. Average nucleotide identity for orthologous genes for Clade 5 strains

	TAL28131	TAL29600	TAL29996	TAL30550	TAL30574
TAL28131	100%	99.83%	99.96%	99.94%	99.88%
TAL29600	99.90%	100%	99.995%	100.00%	99.92%
TAL29996	99.95%	99.996%	100%	99.99%	99.99%
TAL30550	99.96%	99.99%	99.98%	100%	99.96%
TAL30574	99.85%	99.92%	99.99%	99.98%	100%
