# Supplementary material for: Unique growth and morphology properties of Clade 5 Clostridioides difficile strains revealed by single-cell time-lapse microscopy
Source: PLoS Pathog. 2025 May 21;21(5):e1013155. doi: 10.1371/journal.ppat.1013155 (PMC12140426; doi:10.1371/journal.ppat.1013155)
Supplement: S2 Table — (PDF) [file ppat.1013155.s012.pdf]

Predicted mutations

position	mutation	TAL28131	TAL29600	TAL29996	TAL30550	TAL30574	annotation	gene	description
33,367	C→T					100%	G594R (GGA→AGA)	JMW53_00130 ←	AAA family ATPase
47,280	A→C					100%	F32V (TTT→GTT)	JMW53_00190 ←	ABC transporter ATP-binding protein
60,532	A→G					100%	V106A (GTA→GCA)	JMW53_00240 ←	copper-translocating P-type ATPase
72,870	C→T	100%					E221E (GAG→GAA)	JMW53_00290 ←	sel1 repeat family protein
86,141	+66 bp	100%					intergenic (-838/+1969)	JMW53_00355 ← / ← JMW53_00360	CRISPR-associated protein Cas4/CRISPR-associated helicase/endonuclease Cas3
118,805	G→A					100%	Q250Q (CAG→CAA)	JMW53_00495 →	M42 family metallopeptidase
119,649	T→C	100%					I58T (ATT→ACT)	JMW53_00500 →	iron-only hydrogenase system regulator
121,733	T→C				100%		D249D (GAT→GAC)	hydE →	[FeFe] hydrogenase H-cluster radical SAM maturase HydE
138,895	C→A			100%			pseudogene (916/1272 nt)	JMW53_00590 ←	dicarboxylate/amino acid:cation symporter
154,615	C→T	100%					D145N (GAT→AAT)	JMW53_00645 ←	DEAD/DEAH box helicase
162,977	A→C	100%					G146G (GGT→GGG)	tnpB ←	IS200/IS605 family element transposase accessory protein TnpB
162,982	G→T	100%					Q145K (CAA→AAA)	tnpB ←	IS200/IS605 family element transposase accessory protein TnpB
162,987	A→G	100%					V143A (GTA→GCA)	tnpB ←	IS200/IS605 family element transposase accessory protein TnpB
174,989	T→C	100%					S217G (AGT→GGT)	JMW53_00730 ←	2-oxoglutarate oxidoreductase
183,719	C→T	100%					V250I (GTA→ATA)	JMW53_00770 ←	AI-2E family transporter
188,584	G→A	100%					L94L (CTC→CTT)	hflX ←	GTPase HflX
196,933	T→C	100%					pseudogene (355/681 nt)	JMW53_00825 →	hypothetical protein
220,793	T→G					100%	G86G (GGA→GGC)	asnS ←	asparagine--tRNA ligase
222,061	G→A			100%			A526V (GCT→GTT)	JMW53_00940 ←	S8 family peptidase
222,688	G→A					100%	P317L (CCA→CTA)	JMW53_00940 ←	S8 family peptidase
222,814	G→A	100%					A275V (GCA→GTA)	JMW53_00940 ←	S8 family peptidase
271,456	G→T					100%	Q312K (CAA→AAA)	JMW53_01115 ←	sigma 54-interacting transcriptional regulator
287,122	A→G					100%	K53E (AAA→GAA)	JMW53_01205 →	helix-turn-helix transcriptional regulator
330,009	G→T	100%					G86V (GGA→GTA)	JMW53_01390 →	LysR family transcriptional regulator
348,412	A→G	100%					L157L (TTA→CTA)	JMW53_01490 ←	ABC transporter substrate-binding protein
351,165	$(C)_{6\to5}$	100%					intergenic (-13/+245)	JMW53_01510 ← / ← nadC	DsrE family protein/carboxylating nicotinate-nucleotide diphosphorylase
359,596	G→T	100%					A120E (GCA→GAA)	JMW53_01555 ←	hypothetical protein
364,716	$(T)_{6\to5}$			100%			coding (262/1260 nt)	JMW53_01575 ←	aminotransferase class I/II-fold pyridoxal phosphate-dependent enzyme
373,053	A→G	100%					E225E (GAA→GAG)	JMW53_01605 →	alpha-hydroxy-acid oxidizing protein
373,763	Δ4,752 bp			100%				[JMW53_01610]–[JMW53_01635]	[JMW53_01610], JMW53_01615, JMW53_01620, JMW53_01625, JMW53_01630, [JMW53_01635]
375,863	$(T)_{7\to8}$	100%		Δ			intergenic (-266/-412)	JMW53_01620 ← / → JMW53_01625	BlaI/MecI/CopY family transcriptional regulator/transposase
375,898	2 bp→TA	100%		Δ			intergenic (-301/-376)	JMW53_01620 ← / → JMW53_01625	BlaI/MecI/CopY family transcriptional regulator/transposase
375,901	T→G	100%		Δ			intergenic (-304/-374)	JMW53_01620 ← / → JMW53_01625	BlaI/MecI/CopY family transcriptional regulator/transposase
405,339	G→A	100%					G242G (GGC→GGT)	JMW53_01755 ←	NAD(P)-dependent oxidoreductase
411,692	G→T	100%					S80R (AGC→AGA)	JMW53_01785 ←	AraC family transcriptional regulator
414,875	$(T)_{7\to6}$				100%		coding (1117/1137 nt)	JMW53_01800 ←	M20 family metallopeptidase
421,860	C→A	100%					K262N (AAG→AAT)	buk ←	butyrate kinase
449,588	T→A	100%					G248G (GGA→GGT)	JMW53_01975 ←	DevR family CRISPR-associated autoregulator
465,350	C→T	100%					W362* (TGG→TAG)	JMW53_02040 ←	amino acid permease
496,569	A→C	100%					H236Q (CAT→CAG)	JMW53_02185 ←	sporulation-associated two-component system sensor histidine kinase
505,610	A→G	100%	100%	100%	100%	100%	S146P (TCG→CCG)	argH ←	argininosuccinate lyase
514,327	T→G	100%	100%	100%	100%	100%	F260V (TTT→GTT)	JMW53_02250 →	multidrug efflux MFS transporter
520,431	G→A	100%					A39V (GCT→GTT)	JMW53_02275 ←	PRD domain-containing protein
539,169	G→T			100%			E6* (GAA→TAA)	JMW53_02360 →	TetR/AcrR family transcriptional regulator
544,754	2 bp→TT	100%					intergenic (-527/+121)	tnpB ← / ← JMW53_02395	IS200/IS605 family element transposase accessory protein TnpB/alanine--glyoxylate aminotransferase family protein
546,932	A→G	100%					L458L (TTA→CTA)	JMW53_02400 ←	FtsX-like permease family protein
584,856	A→C	100%					N6T (AAC→ACC)	JMW53_02565 →	PTS fructose transporter subunit IIC
584,859	C→G	100%					S7C (TCT→TGT)	JMW53_02565 →	PTS fructose transporter subunit IIC
584,862	T→A	100%					L8H (CTT→CAT)	JMW53_02565 →	PTS fructose transporter subunit IIC
584,865	Δ1 bp	100%					coding (26/1107 nt)	JMW53_02565 →	PTS fructose transporter subunit IIC
591,193	C→T	100%					A40T (GCT→ACT)	JMW53_02575 ←	sensor histidine kinase
627,522	T→G	100%					pseudogene (322/747 nt)	cdtR →	LytTR family response regulator CdtR
648,963	A→G	100%					intergenic (-334/-46)	JMW53_02830 ← / → JMW53_02835	hypothetical protein/PTS sugar transporter subunit IIC
657,646	A→C	100%					W113G (TGG→GGG)	JMW53_02875 ←	NAD(P)H-dependent glycerol-3-phosphate dehydrogenase
674,274	G→A	100%					A208V (GCC→GTC)	JMW53_02970 ←	DUF881 domain-containing protein
725,812	G→A		100%				L276L (CTG→TTG)	JMW53_03170 ←	NAD(P)/FAD-dependent oxidoreductase
736,407	A→T	100%					intergenic (-37/+238)	JMW53_03215 ← / ← JMW53_03220	hypothetical protein/N-acetylmuramoyl-L-alanine amidase
742,451	T→C	100%					pseudogene (400/480 nt)	JMW53_03255 ←	hypothetical protein
757,383	T→C	100%	100%	100%	100%	100%	T175A (ACT→GCT)	JMW53_03310 ←	sugar phosphate isomerase/epimerase
788,619	A→T	100%					N17K (AAT→AAA)	JMW53_03440 ←	hypothetical protein
811,670	T→C	100%					K316E (AAA→GAA)	recJ ←	single-stranded-DNA-specific exonuclease RecJ
825,968	A→G	100%					I292T (ATA→ACA)	JMW53_03590 ←	N-6 DNA methylase
829,966	T→C				100%		M28V (ATG→GTG)	JMW53_03605 ←	N-acetylmuramoyl-L-alanine amidase
845,741	T→A				100%		L233F (TTA→TTT)	JMW53_03675 ←	CDP-glycerol glycerophosphotransferase family protein
846,372	C→T	100%					R23K (AGG→AAG)	JMW53_03675 ←	CDP-glycerol glycerophosphotransferase family protein
856,998	T→C	100%					I269V (ATA→GTA)	JMW53_03720 ←	N-acetylmuramoyl-L-alanine amidase
857,005	A→G	100%					N266N (AAT→AAC)	JMW53_03720 ←	N-acetylmuramoyl-L-alanine amidase
857,029	T→C	100%					V258V (GTA→GTG)	JMW53_03720 ←	N-acetylmuramoyl-L-alanine amidase
857,065	G→A	100%					I246I (ATC→ATT)	JMW53_03720 ←	N-acetylmuramoyl-L-alanine amidase
857,098	T→C	100%					E235E (GAA→GAG)	JMW53_03720 ←	N-acetylmuramoyl-L-alanine amidase
857,104	C→T	100%					K233K (AAG→AAA)	JMW53_03720 ←	N-acetylmuramoyl-L-alanine amidase
857,140	T→C	100%					L221L (TTA→TTG)	JMW53_03720 ←	N-acetylmuramoyl-L-alanine amidase
857,182	G→A	100%					I207I (ATC→ATT)	JMW53_03720 ←	N-acetylmuramoyl-L-alanine amidase
857,229	2 bp→CT	100%					coding (573-574/2025 nt)	JMW53_03720 ←	N-acetylmuramoyl-L-alanine amidase
857,283	2 bp→CT	100%					coding (519-520/2025 nt)	JMW53_03720 ←	N-acetylmuramoyl-L-alanine amidase
857,293	T→C	100%					K170K (AAA→AAG)	JMW53_03720 ←	N-acetylmuramoyl-L-alanine amidase
857,334	C→T	100%					V157I (GTA→ATA)	JMW53_03720 ←	N-acetylmuramoyl-L-alanine amidase
857,401	T→C	100%					G134G (GGA→GGG)	JMW53_03720 ←	N-acetylmuramoyl-L-alanine amidase
857,416	G→A	100%					D129D (GAC→GAT)	JMW53_03720 ←	N-acetylmuramoyl-L-alanine amidase
857,425	G→A	100%					N126N (AAC→AAT)	JMW53_03720 ←	N-acetylmuramoyl-L-alanine amidase
857,449	G→A	100%					G118G (GGC→GGT)	JMW53_03720 ←	N-acetylmuramoyl-L-alanine amidase
857,509	G→A	100%					N98N (AAC→AAT)	JMW53_03720 ←	N-acetylmuramoyl-L-alanine amidase
857,695	G→A	100%					N36N (AAC→AAT)	JMW53_03720 ←	N-acetylmuramoyl-L-alanine amidase
857,766	C→A	100%					V13L (GTG→TTG)	JMW53_03720 ←	N-acetylmuramoyl-L-alanine amidase
857,817	Δ1 bp	100%					intergenic (-15/+240)	JMW53_03720 ← / ← JMW53_03725	N-acetylmuramoyl-L-alanine amidase/DUF5009 domain-containing protein
857,826	2 bp→TT	100%					intergenic (-24/+230)	JMW53_03720 ← / ← JMW53_03725	N-acetylmuramoyl-L-alanine amidase/DUF5009 domain-containing protein
857,924	$(A)_{6\to5}$					100%	intergenic (-122/+133)	JMW53_03720 ← / ← JMW53_03725	N-acetylmuramoyl-L-alanine amidase/DUF5009 domain-containing protein
857,983	G→A	100%					intergenic (-181/+74)	JMW53_03720 ← / ← JMW53_03725	N-acetylmuramoyl-L-alanine amidase/DUF5009 domain-containing protein
858,023	C→T	100%					intergenic (-221/+34)	JMW53_03720 ← / ← JMW53_03725	N-acetylmuramoyl-L-alanine amidase/DUF5009 domain-containing protein
858,027	A→T	100%					intergenic (-225/+30)	JMW53_03720 ← / ← JMW53_03725	N-acetylmuramoyl-L-alanine amidase/DUF5009 domain-containing protein
858,039	G→T	100%					intergenic (-237/+18)	JMW53_03720 ← / ← JMW53_03725	N-acetylmuramoyl-L-alanine amidase/DUF5009 domain-containing protein
858,066	G→A	100%					I473I (ATC→ATT)	JMW53_03725 ←	DUF5009 domain-containing protein
858,084	A→G	100%					Y467Y (TAT→TAC)	JMW53_03725 ←	DUF5009 domain-containing protein
858,090	A→T	100%					I465I (ATT→ATA)	JMW53_03725 ←	DUF5009 domain-containing protein
858,129	A→C	100%					S452S (TCT→TCG)	JMW53_03725 ←	DUF5009 domain-containing protein
858,168	A→T	100%					T439T (ACT→ACA)	JMW53_03725 ←	DUF5009 domain-containing protein
858,225	T→C	100%					T420T (ACA→ACG)	JMW53_03725 ←	DUF5009 domain-containing protein
858,239	2 bp→CA	100%					coding (1245-1246/1428 nt)	JMW53_03725 ←	DUF5009 domain-containing protein
858,255	G→A	100%					S410S (AGC→AGT)	JMW53_03725 ←	DUF5009 domain-containing protein
858,258	T→C	100%					L409L (TTA→TTG)	JMW53_03725 ←	DUF5009 domain-containing protein
858,267	G→A	100%					L406L (CTC→CTT)	JMW53_03725 ←	DUF5009 domain-containing protein
858,270	A→G	100%					C405C (TGT→TGC)	JMW53_03725 ←	DUF5009 domain-containing protein
858,315	G→A	100%					T390T (ACC→ACT)	JMW53_03725 ←	DUF5009 domain-containing protein
858,345	A→G	100%					C380C (TGT→TGC)	JMW53_03725 ←	DUF5009 domain-containing protein
858,374	T→C	100%					I371V (ATA→GTA)	JMW53_03725 ←	DUF5009 domain-containing protein
858,384	T→G	100%					G367G (GGA→GGC)	JMW53_03725 ←	DUF5009 domain-containing protein
858,429	A→G	100%					F352F (TTT→TTC)	JMW53_03725 ←	DUF5009 domain-containing protein
858,450	T→G	100%					I345I (ATA→ATC)	JMW53_03725 ←	DUF5009 domain-containing protein
858,468	T→C	100%					L339L (TTA→TTG)	JMW53_03725 ←	DUF5009 domain-containing protein
858,470	2 bp→GG	100%					coding (1014-1015/1428 nt)	JMW53_03725 ←	DUF5009 domain-containing protein
858,474	A→T	100%					I337I (ATT→ATA)	JMW53_03725 ←	DUF5009 domain-containing protein
858,477	A→G	100%					S336S (AGT→AGC)	JMW53_03725 ←	DUF5009 domain-containing protein
858,486	T→C	100%					L333M (TTA→CTG)	JMW53_03725 ←	DUF5009 domain-containing protein
858,488	A→G	100%					L333M (TTA→CTG)	JMW53_03725 ←	DUF5009 domain-containing protein
858,510	C→T	100%					E325E (GAG→GAA)	JMW53_03725 ←	DUF5009 domain-containing protein
858,534	C→T	100%					V317V (GTG→GTA)	JMW53_03725 ←	DUF5009 domain-containing protein
858,546	2 bp→AG	100%					coding (938-939/1428 nt)	JMW53_03725 ←	DUF5009 domain-containing protein
858,561	T→A	100%					G308G (GGA→GGT)	JMW53_03725 ←	DUF5009 domain-containing protein
858,603	C→T	100%					E294E (GAG→GAA)	JMW53_03725 ←	DUF5009 domain-containing protein
858,609	A→T	100%					D292E (GAT→GAA)	JMW53_03725 ←	DUF5009 domain-containing protein
858,654	T→C	100%					Q277Q (CAA→CAG)	JMW53_03725 ←	DUF5009 domain-containing protein
858,681	C→T	100%					G268G (GGG→GGA)	JMW53_03725 ←	DUF5009 domain-containing protein
858,698	C→A	100%					V263L (GTA→TTA)	JMW53_03725 ←	DUF5009 domain-containing protein
858,705	A→G	100%					Y260Y (TAT→TAC)	JMW53_03725 ←	DUF5009 domain-containing protein
858,708	T→C	100%					G259G (GGA→GGG)	JMW53_03725 ←	DUF5009 domain-containing protein
858,711	T→C	100%					V258V (GTA→GTG)	JMW53_03725 ←	DUF5009 domain-containing protein
858,724	G→A	100%					A254V (GCA→GTA)	JMW53_03725 ←	DUF5009 domain-containing protein
858,746	T→G	100%					I247L (ATT→CTT)	JMW53_03725 ←	DUF5009 domain-containing protein
858,754	A→G	100%					V244A (GTT→GCT)	JMW53_03725 ←	DUF5009 domain-containing protein
858,758	2 bp→TG	100%					coding (726-727/1428 nt)	JMW53_03725 ←	DUF5009 domain-containing protein
858,764	T→C	100%					R241G (AGA→GGA)	JMW53_03725 ←	DUF5009 domain-containing protein
858,801	A→G	100%					T228T (ACT→ACC)	JMW53_03725 ←	DUF5009 domain-containing protein
858,804	C→A	100%					V227V (GTG→GTT)	JMW53_03725 ←	DUF5009 domain-containing protein
858,875	G→A	100%					L204L (CTA→TTA)	JMW53_03725 ←	DUF5009 domain-containing protein
858,909	G→A	100%					I192I (ATC→ATT)	JMW53_03725 ←	DUF5009 domain-containing protein
858,942	T→A	100%					L181L (TTA→CTT)	JMW53_03725 ←	DUF5009 domain-containing protein
858,944	A→G	100%					L181L (TTA→CTT)	JMW53_03725 ←	DUF5009 domain-containing protein
858,948	A→G	100%					S179S (TCT→TCC)	JMW53_03725 ←	DUF5009 domain-containing protein
858,969	A→G	100%					S172S (AGT→AGC)	JMW53_03725 ←	DUF5009 domain-containing protein
858,978	A→G	100%					S169S (TCA→TCC)	JMW53_03725 ←	DUF5009 domain-containing protein
859,002	G→C	100%					S161S (TCC→TCG)	JMW53_03725 ←	DUF5009 domain-containing protein
859,017	A→C	100%					P156P (CCT→CCG)	JMW53_03725 ←	DUF5009 domain-containing protein
859,023	T→C	100%					A154A (GCA→GCG)	JMW53_03725 ←	DUF5009 domain-containing protein
859,044	A→G	100%					G147G (GGT→GGC)	JMW53_03725 ←	DUF5009 domain-containing protein
859,065	A→T	100%					L140L (CTT→CTA)	JMW53_03725 ←	DUF5009 domain-containing protein
859,092	C→T	100%					G131G (GGG→GGA)	JMW53_03725 ←	DUF5009 domain-containing protein
859,107	A→G	100%					V126V (GTT→GTC)	JMW53_03725 ←	DUF5009 domain-containing protein
859,143	A→G	100%					D114D (GAT→GAC)	JMW53_03725 ←	DUF5009 domain-containing protein
859,161	A→G	100%					S108S (AGT→AGC)	JMW53_03725 ←	DUF5009 domain-containing protein
859,169	C→T	100%					V106M (GTG→ATG)	JMW53_03725 ←	DUF5009 domain-containing protein
859,172	A→G	100%					F105L (TTT→CTT)	JMW53_03725 ←	DUF5009 domain-containing protein
859,184	T→C	100%					I101V (ATA→GTA)	JMW53_03725 ←	DUF5009 domain-containing protein
859,187	T→C	100%					S100G (AGT→GGT)	JMW53_03725 ←	DUF5009 domain-containing protein
859,236	G→T	?			100%		S83R (AGC→AGA)	JMW53_03725 ←	DUF5009 domain-containing protein
859,445	T→C	100%					S14G (AGT→GGT)	JMW53_03725 ←	DUF5009 domain-containing protein
859,451	G→T	100%					H12N (CAT→AAT)	JMW53_03725 ←	DUF5009 domain-containing protein
859,471	A→G	100%					I5T (ATT→ACT)	JMW53_03725 ←	DUF5009 domain-containing protein
859,476	G→A	100%					S3S (AGC→AGT)	JMW53_03725 ←	DUF5009 domain-containing protein
859,498	G→A	100%					intergenic (-14/+121)	JMW53_03725 ← / ← JMW53_03730	DUF5009 domain-containing protein/cell wall-binding protein Cwp5
859,500	T→C	100%					intergenic (-16/+119)	JMW53_03725 ← / ← JMW53_03730	DUF5009 domain-containing protein/cell wall-binding protein Cwp5
859,502	T→C	100%					intergenic (-18/+117)	JMW53_03725 ← / ← JMW53_03730	DUF5009 domain-containing protein/cell wall-binding protein Cwp5
859,506	T→C	100%					intergenic (-22/+113)	JMW53_03725 ← / ← JMW53_03730	DUF5009 domain-containing protein/cell wall-binding protein Cwp5
859,509	C→A	100%					intergenic (-25/+110)	JMW53_03725 ← / ← JMW53_03730	DUF5009 domain-containing protein/cell wall-binding protein Cwp5
859,516	T→C	100%					intergenic (-32/+103)	JMW53_03725 ← / ← JMW53_03730	DUF5009 domain-containing protein/cell wall-binding protein Cwp5
859,518	G→A	100%					intergenic (-34/+101)	JMW53_03725 ← / ← JMW53_03730	DUF5009 domain-containing protein/cell wall-binding protein Cwp5
859,521	2 bp→TC	100%					intergenic (-37/+97)	JMW53_03725 ← / ← JMW53_03730	DUF5009 domain-containing protein/cell wall-binding protein Cwp5
859,524	T→C	100%					intergenic (-40/+95)	JMW53_03725 ← / ← JMW53_03730	DUF5009 domain-containing protein/cell wall-binding protein Cwp5
859,526	T→C	100%					intergenic (-42/+93)	JMW53_03725 ← / ← JMW53_03730	DUF5009 domain-containing protein/cell wall-binding protein Cwp5
859,532	G→T	100%					intergenic (-48/+87)	JMW53_03725 ← / ← JMW53_03730	DUF5009 domain-containing protein/cell wall-binding protein Cwp5
859,540	C→T	100%					intergenic (-56/+79)	JMW53_03725 ← / ← JMW53_03730	DUF5009 domain-containing protein/cell wall-binding protein Cwp5
859,547	A→G	100%					intergenic (-63/+72)	JMW53_03725 ← / ← JMW53_03730	DUF5009 domain-containing protein/cell wall-binding protein Cwp5
859,549	C→A	100%					intergenic (-65/+70)	JMW53_03725 ← / ← JMW53_03730	DUF5009 domain-containing protein/cell wall-binding protein Cwp5
859,556	C→T	100%					intergenic (-72/+63)	JMW53_03725 ← / ← JMW53_03730	DUF5009 domain-containing protein/cell wall-binding protein Cwp5
859,558	C→T	100%					intergenic (-74/+61)	JMW53_03725 ← / ← JMW53_03730	DUF5009 domain-containing protein/cell wall-binding protein Cwp5
859,567	A→T	100%					intergenic (-83/+52)	JMW53_03725 ← / ← JMW53_03730	DUF5009 domain-containing protein/cell wall-binding protein Cwp5
859,573	C→A	100%					intergenic (-89/+46)	JMW53_03725 ← / ← JMW53_03730	DUF5009 domain-containing protein/cell wall-binding protein Cwp5
859,580	Δ1 bp	100%					intergenic (-96/+39)	JMW53_03725 ← / ← JMW53_03730	DUF5009 domain-containing protein/cell wall-binding protein Cwp5
859,582	T→C	100%					intergenic (-98/+37)	JMW53_03725 ← / ← JMW53_03730	DUF5009 domain-containing protein/cell wall-binding protein Cwp5
859,587	C→A	100%					intergenic (-103/+32)	JMW53_03725 ← / ← JMW53_03730	DUF5009 domain-containing protein/cell wall-binding protein Cwp5
859,591	C→T	100%					intergenic (-107/+28)	JMW53_03725 ← / ← JMW53_03730	DUF5009 domain-containing protein/cell wall-binding protein Cwp5
859,597	G→A	100%					intergenic (-113/+22)	JMW53_03725 ← / ← JMW53_03730	DUF5009 domain-containing protein/cell wall-binding protein Cwp5
859,612	Δ2 bp	100%					intergenic (-128/+6)	JMW53_03725 ← / ← JMW53_03730	DUF5009 domain-containing protein/cell wall-binding protein Cwp5
859,618	T→C	100%					intergenic (-134/+1)	JMW53_03725 ← / ← JMW53_03730	DUF5009 domain-containing protein/cell wall-binding protein Cwp5
859,643	T→C	100%					G518G (GGA→GGG)	JMW53_03730 ←	cell wall-binding protein Cwp5
859,661	A→G	100%					D512D (GAT→GAC)	JMW53_03730 ←	cell wall-binding protein Cwp5
859,678	T→C	100%					I507V (ATC→GTC)	JMW53_03730 ←	cell wall-binding protein Cwp5
859,718	A→T	100%					V493V (GTT→GTA)	JMW53_03730 ←	cell wall-binding protein Cwp5
859,721	C→T	100%					K492K (AAG→AAA)	JMW53_03730 ←	cell wall-binding protein Cwp5
859,736	C→T	100%					E487E (GAG→GAA)	JMW53_03730 ←	cell wall-binding protein Cwp5
859,742	C→A	100%					Q485H (CAG→CAT)	JMW53_03730 ←	cell wall-binding protein Cwp5
859,748	A→G	100%					V483V (GTT→GTC)	JMW53_03730 ←	cell wall-binding protein Cwp5
859,751	T→C	100%					K482K (AAA→AAG)	JMW53_03730 ←	cell wall-binding protein Cwp5
859,766	A→C	100%					G477G (GGT→GGG)	JMW53_03730 ←	cell wall-binding protein Cwp5
859,916	G→A	100%					D427D (GAC→GAT)	JMW53_03730 ←	cell wall-binding protein Cwp5
859,934	2 bp→AC	100%					coding (1262-1263/1578 nt)	JMW53_03730 ←	cell wall-binding protein Cwp5
859,937	G→T	100%					I420I (ATC→ATA)	JMW53_03730 ←	cell wall-binding protein Cwp5
859,940	T→C	100%					T419T (ACA→ACG)	JMW53_03730 ←	cell wall-binding protein Cwp5
860,045	T→C	100%					K384K (AAA→AAG)	JMW53_03730 ←	cell wall-binding protein Cwp5
860,057	T→A	100%					G380G (GGA→GGT)	JMW53_03730 ←	cell wall-binding protein Cwp5
860,060	T→C	100%					K379K (AAA→AAG)	JMW53_03730 ←	cell wall-binding protein Cwp5
860,090	C→T	100%					V369V (GTG→GTA)	JMW53_03730 ←	cell wall-binding protein Cwp5
860,119	2 bp→TC	100%					coding (1077-1078/1578 nt)	JMW53_03730 ←	cell wall-binding protein Cwp5
860,123	T→A	100%					K358N (AAA→AAT)	JMW53_03730 ←	cell wall-binding protein Cwp5
860,183	C→T	100%					E338E (GAG→GAA)	JMW53_03730 ←	cell wall-binding protein Cwp5
860,204	C→T	100%					V331V (GTG→GTA)	JMW53_03730 ←	cell wall-binding protein Cwp5
860,221	2 bp→GG	100%					coding (975-976/1578 nt)	JMW53_03730 ←	cell wall-binding protein Cwp5
860,231	C→T	100%					E322E (GAG→GAA)	JMW53_03730 ←	cell wall-binding protein Cwp5
860,306	C→T	100%					R297R (AGG→AGA)	JMW53_03730 ←	cell wall-binding protein Cwp5
860,330	G→A	100%					G289G (GGC→GGT)	JMW53_03730 ←	cell wall-binding protein Cwp5
860,360	T→G	100%					A279A (GCA→GCC)	JMW53_03730 ←	cell wall-binding protein Cwp5
860,368	2 bp→AA	100%					coding (828-829/1578 nt)	JMW53_03730 ←	cell wall-binding protein Cwp5
860,378	T→A	100%					A273A (GCA→GCT)	JMW53_03730 ←	cell wall-binding protein Cwp5
860,387	G→A	100%					D270D (GAC→GAT)	JMW53_03730 ←	cell wall-binding protein Cwp5
860,393	C→T	100%					L268L (CTG→CTA)	JMW53_03730 ←	cell wall-binding protein Cwp5
860,396	T→C	100%					Q267Q (CAA→CAG)	JMW53_03730 ←	cell wall-binding protein Cwp5
860,402	T→C	100%					E265E (GAA→GAG)	JMW53_03730 ←	cell wall-binding protein Cwp5
860,414	T→A	100%					G261G (GGA→GGT)	JMW53_03730 ←	cell wall-binding protein Cwp5
860,426	T→C	100%					V257V (GTA→GTG)	JMW53_03730 ←	cell wall-binding protein Cwp5
860,434	G→A	100%					L255L (CTA→TTA)	JMW53_03730 ←	cell wall-binding protein Cwp5
860,441	T→C	100%					L252L (TTA→TTG)	JMW53_03730 ←	cell wall-binding protein Cwp5
860,445	C→T	100%					S251N (AGT→AAT)	JMW53_03730 ←	cell wall-binding protein Cwp5
860,507	T→C	100%					L230L (TTA→TTG)	JMW53_03730 ←	cell wall-binding protein Cwp5
860,552	T→A	100%					S215S (TCA→TCT)	JMW53_03730 ←	cell wall-binding protein Cwp5
860,561	C→A	100%					G212G (GGG→GGT)	JMW53_03730 ←	cell wall-binding protein Cwp5
860,588	T→C	100%					K203K (AAA→AAG)	JMW53_03730 ←	cell wall-binding protein Cwp5
860,612	A→G	100%					F195F (TTT→TTC)	JMW53_03730 ←	cell wall-binding protein Cwp5
860,624	T→C	100%					G191G (GGA→GGG)	JMW53_03730 ←	cell wall-binding protein Cwp5
860,642	C→T	100%					L185L (TTG→TTA)	JMW53_03730 ←	cell wall-binding protein Cwp5
860,701	G→A	100%					L166L (CTG→TTG)	JMW53_03730 ←	cell wall-binding protein Cwp5
860,705	T→G	100%					K164N (AAA→AAC)	JMW53_03730 ←	cell wall-binding protein Cwp5
860,732	T→C	100%					K155K (AAA→AAG)	JMW53_03730 ←	cell wall-binding protein Cwp5
860,771	T→C	100%					L142L (CTA→CTG)	JMW53_03730 ←	cell wall-binding protein Cwp5
860,792	C→T	100%					K135K (AAG→AAA)	JMW53_03730 ←	cell wall-binding protein Cwp5
860,921	T→C	100%					R92R (AGA→AGG)	JMW53_03730 ←	cell wall-binding protein Cwp5
860,960	T→A	100%					A79A (GCA→GCT)	JMW53_03730 ←	cell wall-binding protein Cwp5
860,972	C→T	100%					K75K (AAG→AAA)	JMW53_03730 ←	cell wall-binding protein Cwp5
860,987	A→G	100%					A70A (GCT→GCC)	JMW53_03730 ←	cell wall-binding protein Cwp5
861,023	T→C	100%					L58L (TTA→TTG)	JMW53_03730 ←	cell wall-binding protein Cwp5
861,044	A→G	100%					S51S (AGT→AGC)	JMW53_03730 ←	cell wall-binding protein Cwp5
861,097	C→T	100%					V34I (GTA→ATA)	JMW53_03730 ←	cell wall-binding protein Cwp5
861,201	A→C	100%					intergenic (-5/+298)	JMW53_03730 ← / ← JMW53_03735	cell wall-binding protein Cwp5/cell wall-binding cysteine protease Cwp84
861,206	T→C	100%					intergenic (-10/+293)	JMW53_03730 ← / ← JMW53_03735	cell wall-binding protein Cwp5/cell wall-binding cysteine protease Cwp84
861,301	A→G	100%					intergenic (-105/+198)	JMW53_03730 ← / ← JMW53_03735	cell wall-binding protein Cwp5/cell wall-binding cysteine protease Cwp84
861,304	2 bp→AA	100%					intergenic (-108/+194)	JMW53_03730 ← / ← JMW53_03735	cell wall-binding protein Cwp5/cell wall-binding cysteine protease Cwp84
861,316	+A	100%					intergenic (-120/+183)	JMW53_03730 ← / ← JMW53_03735	cell wall-binding protein Cwp5/cell wall-binding cysteine protease Cwp84
861,323	C→T	100%					intergenic (-127/+176)	JMW53_03730 ← / ← JMW53_03735	cell wall-binding protein Cwp5/cell wall-binding cysteine protease Cwp84
861,326	T→G	100%					intergenic (-130/+173)	JMW53_03730 ← / ← JMW53_03735	cell wall-binding protein Cwp5/cell wall-binding cysteine protease Cwp84
861,335	2 bp→TA	100%					intergenic (-139/+163)	JMW53_03730 ← / ← JMW53_03735	cell wall-binding protein Cwp5/cell wall-binding cysteine protease Cwp84
861,339	T→C	100%					intergenic (-143/+160)	JMW53_03730 ← / ← JMW53_03735	cell wall-binding protein Cwp5/cell wall-binding cysteine protease Cwp84
861,343	G→A	100%					intergenic (-147/+156)	JMW53_03730 ← / ← JMW53_03735	cell wall-binding protein Cwp5/cell wall-binding cysteine protease Cwp84
861,347	T→C	100%					intergenic (-151/+152)	JMW53_03730 ← / ← JMW53_03735	cell wall-binding protein Cwp5/cell wall-binding cysteine protease Cwp84
861,349	A→C	100%					intergenic (-153/+150)	JMW53_03730 ← / ← JMW53_03735	cell wall-binding protein Cwp5/cell wall-binding cysteine protease Cwp84
861,365	A→T	100%					intergenic (-169/+134)	JMW53_03730 ← / ← JMW53_03735	cell wall-binding protein Cwp5/cell wall-binding cysteine protease Cwp84
861,383	G→A	100%					intergenic (-187/+116)	JMW53_03730 ← / ← JMW53_03735	cell wall-binding protein Cwp5/cell wall-binding cysteine protease Cwp84
861,391	G→A	100%					intergenic (-195/+108)	JMW53_03730 ← / ← JMW53_03735	cell wall-binding protein Cwp5/cell wall-binding cysteine protease Cwp84
861,401	T→C	100%					intergenic (-205/+98)	JMW53_03730 ← / ← JMW53_03735	cell wall-binding protein Cwp5/cell wall-binding cysteine protease Cwp84
861,413	C→T	100%					intergenic (-217/+86)	JMW53_03730 ← / ← JMW53_03735	cell wall-binding protein Cwp5/cell wall-binding cysteine protease Cwp84
861,416	C→T	100%					intergenic (-220/+83)	JMW53_03730 ← / ← JMW53_03735	cell wall-binding protein Cwp5/cell wall-binding cysteine protease Cwp84
861,431	T→C	100%					intergenic (-235/+68)	JMW53_03730 ← / ← JMW53_03735	cell wall-binding protein Cwp5/cell wall-binding cysteine protease Cwp84
861,433	T→C	100%					intergenic (-237/+66)	JMW53_03730 ← / ← JMW53_03735	cell wall-binding protein Cwp5/cell wall-binding cysteine protease Cwp84
861,493	T→C	100%					intergenic (-297/+6)	JMW53_03730 ← / ← JMW53_03735	cell wall-binding protein Cwp5/cell wall-binding cysteine protease Cwp84
861,596	G→T	100%					T772N (ACT→AAT)	JMW53_03735 ←	cell wall-binding cysteine protease Cwp84
861,675	G→A	100%					L746L (CTA→TTA)	JMW53_03735 ←	cell wall-binding cysteine protease Cwp84
861,688	A→G	100%					D741D (GAT→GAC)	JMW53_03735 ←	cell wall-binding cysteine protease Cwp84
861,699	G→T	100%					R738S (CGT→AGT)	JMW53_03735 ←	cell wall-binding cysteine protease Cwp84
861,743	C→T	100%					S723N (AGT→AAT)	JMW53_03735 ←	cell wall-binding cysteine protease Cwp84
861,748	G→A	100%					Y721Y (TAC→TAT)	JMW53_03735 ←	cell wall-binding cysteine protease Cwp84
861,751	G→A	100%					F720F (TTC→TTT)	JMW53_03735 ←	cell wall-binding cysteine protease Cwp84
861,771	C→A	100%					A714S (GCT→TCT)	JMW53_03735 ←	cell wall-binding cysteine protease Cwp84
861,955	A→G	100%					D652D (GAT→GAC)	JMW53_03735 ←	cell wall-binding cysteine protease Cwp84
862,021	T→C	100%					Q630Q (CAA→CAG)	JMW53_03735 ←	cell wall-binding cysteine protease Cwp84
862,102	T→C	100%					V603V (GTA→GTG)	JMW53_03735 ←	cell wall-binding cysteine protease Cwp84
862,120	G→A	100%					S597S (AGC→AGT)	JMW53_03735 ←	cell wall-binding cysteine protease Cwp84
862,131	C→T	100%					A594T (GCT→ACT)	JMW53_03735 ←	cell wall-binding cysteine protease Cwp84
862,137	C→T	100%					V592I (GTA→ATA)	JMW53_03735 ←	cell wall-binding cysteine protease Cwp84
862,198	T→C	100%					L571L (TTA→TTG)	JMW53_03735 ←	cell wall-binding cysteine protease Cwp84
862,206	A→C	100%					S569A (TCA→GCA)	JMW53_03735 ←	cell wall-binding cysteine protease Cwp84
862,258	G→T	100%					I551I (ATC→ATA)	JMW53_03735 ←	cell wall-binding cysteine protease Cwp84
862,262	G→A	100%					A550V (GCA→GTA)	JMW53_03735 ←	cell wall-binding cysteine protease Cwp84
862,294	C→T	100%					V539V (GTG→GTA)	JMW53_03735 ←	cell wall-binding cysteine protease Cwp84
862,351	A→G	100%					S520S (AGT→AGC)	JMW53_03735 ←	cell wall-binding cysteine protease Cwp84
862,537	T→C	100%					E458E (GAA→GAG)	JMW53_03735 ←	cell wall-binding cysteine protease Cwp84
862,545	A→G	100%					L456L (TTA→CTA)	JMW53_03735 ←	cell wall-binding cysteine protease Cwp84
862,553	G→C	100%					A453G (GCA→GGA)	JMW53_03735 ←	cell wall-binding cysteine protease Cwp84
862,660	A→G	100%					N417N (AAT→AAC)	JMW53_03735 ←	cell wall-binding cysteine protease Cwp84
862,711	T→G	100%					K400N (AAA→AAC)	JMW53_03735 ←	cell wall-binding cysteine protease Cwp84
862,726	A→G	100%					N395N (AAT→AAC)	JMW53_03735 ←	cell wall-binding cysteine protease Cwp84
862,912	A→G	100%					D333D (GAT→GAC)	JMW53_03735 ←	cell wall-binding cysteine protease Cwp84
862,968	G→A	100%					L315L (CTA→TTA)	JMW53_03735 ←	cell wall-binding cysteine protease Cwp84
863,017	C→T	100%					E298E (GAG→GAA)	JMW53_03735 ←	cell wall-binding cysteine protease Cwp84
863,116	C→A	100%					A265A (GCG→GCT)	JMW53_03735 ←	cell wall-binding cysteine protease Cwp84
863,239	T→C	100%					K224K (AAA→AAG)	JMW53_03735 ←	cell wall-binding cysteine protease Cwp84
863,293	T→A	100%					T206T (ACA→ACT)	JMW53_03735 ←	cell wall-binding cysteine protease Cwp84
863,308	C→T	100%					M201I (ATG→ATA)	JMW53_03735 ←	cell wall-binding cysteine protease Cwp84
863,311	A→G	100%					N200N (AAT→AAC)	JMW53_03735 ←	cell wall-binding cysteine protease Cwp84
863,335	C→T	100%					A192A (GCG→GCA)	JMW53_03735 ←	cell wall-binding cysteine protease Cwp84
863,374	C→T	100%					K179K (AAG→AAA)	JMW53_03735 ←	cell wall-binding cysteine protease Cwp84
863,425	A→C	100%					G162G (GGT→GGG)	JMW53_03735 ←	cell wall-binding cysteine protease Cwp84
863,456	T→G	100%					K152T (AAA→ACA)	JMW53_03735 ←	cell wall-binding cysteine protease Cwp84
863,504	G→A	100%					T136M (ACG→ATG)	JMW53_03735 ←	cell wall-binding cysteine protease Cwp84
863,520	A→G	100%					L131L (TTA→CTA)	JMW53_03735 ←	cell wall-binding cysteine protease Cwp84
863,536	T→C	100%					L125L (TTA→TTG)	JMW53_03735 ←	cell wall-binding cysteine protease Cwp84
863,548	A→C	100%					G121G (GGT→GGG)	JMW53_03735 ←	cell wall-binding cysteine protease Cwp84
863,641	G→A	100%					Y90Y (TAC→TAT)	JMW53_03735 ←	cell wall-binding cysteine protease Cwp84
863,646	A→C	100%					S89A (TCA→GCA)	JMW53_03735 ←	cell wall-binding cysteine protease Cwp84
863,653	A→G	100%					L86L (CTT→CTC)	JMW53_03735 ←	cell wall-binding cysteine protease Cwp84
863,667	T→C	100%					S82G (AGT→GGT)	JMW53_03735 ←	cell wall-binding cysteine protease Cwp84
863,671	2 bp→GT	100%					coding (239-240/2412 nt)	JMW53_03735 ←	cell wall-binding cysteine protease Cwp84
863,731	T→C	100%					T60T (ACA→ACG)	JMW53_03735 ←	cell wall-binding cysteine protease Cwp84
863,770	G→T	100%					S47G (AGC→GGA)	JMW53_03735 ←	cell wall-binding cysteine protease Cwp84
863,772	T→C	100%					S47G (AGC→GGA)	JMW53_03735 ←	cell wall-binding cysteine protease Cwp84
863,814	T→G	100%					N33H (AAT→CAT)	JMW53_03735 ←	cell wall-binding cysteine protease Cwp84
863,899	G→A	100%					Y4Y (TAC→TAT)	JMW53_03735 ←	cell wall-binding cysteine protease Cwp84
864,002	G→A	100%					intergenic (-92/-136)	JMW53_03735 ← / → JMW53_03740	cell wall-binding cysteine protease Cwp84/GtrA family protein
864,018	A→G	100%					intergenic (-108/-120)	JMW53_03735 ← / → JMW53_03740	cell wall-binding cysteine protease Cwp84/GtrA family protein
864,024	G→A	100%					intergenic (-114/-114)	JMW53_03735 ← / → JMW53_03740	cell wall-binding cysteine protease Cwp84/GtrA family protein
864,064	G→A	100%					intergenic (-154/-74)	JMW53_03735 ← / → JMW53_03740	cell wall-binding cysteine protease Cwp84/GtrA family protein
864,104	A→G	100%					intergenic (-194/-34)	JMW53_03735 ← / → JMW53_03740	cell wall-binding cysteine protease Cwp84/GtrA family protein
864,157	2 bp→GA	100%					coding (20-21/402 nt)	JMW53_03740 →	GtrA family protein
864,241	C→T	100%					S35L (TCA→TTA)	JMW53_03740 →	GtrA family protein
864,264	G→A	100%					A43T (GCT→ACT)	JMW53_03740 →	GtrA family protein
864,325	2 bp→GG	100%					coding (188-189/402 nt)	JMW53_03740 →	GtrA family protein
864,336	A→G	100%					I67V (ATC→GTT)	JMW53_03740 →	GtrA family protein
864,338	C→T	100%					I67V (ATC→GTT)	JMW53_03740 →	GtrA family protein
864,341	2 bp→GA	100%					coding (204-205/402 nt)	JMW53_03740 →	GtrA family protein
864,353	G→A	100%					L72L (TTG→TTA)	JMW53_03740 →	GtrA family protein
864,368	T→G	100%					F77L (TTT→TTG)	JMW53_03740 →	GtrA family protein
864,371	T→C	100%					S78S (TCT→TCC)	JMW53_03740 →	GtrA family protein
864,380	T→C	100%					S81S (AGT→AGC)	JMW53_03740 →	GtrA family protein
864,386	G→A	100%					R83R (AGG→AGA)	JMW53_03740 →	GtrA family protein
864,458	T→A	100%					I107I (ATT→ATA)	JMW53_03740 →	GtrA family protein
864,461	A→G	100%					V108V (GTA→GTG)	JMW53_03740 →	GtrA family protein
864,467	A→C	100%					V110V (GTA→GTC)	JMW53_03740 →	GtrA family protein
864,480	G→A	100%					V115I (GTT→ATT)	JMW53_03740 →	GtrA family protein
864,503	G→A	100%					L122L (TTG→TTA)	JMW53_03740 →	GtrA family protein
864,512	G→A	100%					K125K (AAG→AAA)	JMW53_03740 →	GtrA family protein
864,525	+G	100%					coding (388/402 nt)	JMW53_03740 →	GtrA family protein
866,098	T→C	100%					L107L (TTA→TTG)	JMW53_03745 ←	cell wall-binding repeat-containing protein
866,119	A→T	100%					L100L (CTT→CTA)	JMW53_03745 ←	cell wall-binding repeat-containing protein
866,122	T→C	100%					R99R (AGA→AGG)	JMW53_03745 ←	cell wall-binding repeat-containing protein
866,125	T→C	100%					K98K (AAA→AAG)	JMW53_03745 ←	cell wall-binding repeat-containing protein
866,135	G→A	100%					A95V (GCA→GTA)	JMW53_03745 ←	cell wall-binding repeat-containing protein
866,140	G→C	100%					T93T (ACC→ACG)	JMW53_03745 ←	cell wall-binding repeat-containing protein
866,147	2 bp→TC	100%					coding (271-272/1833 nt)	JMW53_03745 ←	cell wall-binding repeat-containing protein
866,179	T→C	100%					P80P (CCA→CCG)	JMW53_03745 ←	cell wall-binding repeat-containing protein
866,203	T→C	100%					P72P (CCA→CCG)	JMW53_03745 ←	cell wall-binding repeat-containing protein
866,230	G→T	100%					S63S (TCC→TCA)	JMW53_03745 ←	cell wall-binding repeat-containing protein
866,236	G→A	100%					D61D (GAC→GAT)	JMW53_03745 ←	cell wall-binding repeat-containing protein
866,247	2 bp→GC	100%					coding (171-172/1833 nt)	JMW53_03745 ←	cell wall-binding repeat-containing protein
866,254	2 bp→AC	100%					coding (164-165/1833 nt)	JMW53_03745 ←	cell wall-binding repeat-containing protein
866,257	A→T	100%					D54E (GAT→GAA)	JMW53_03745 ←	cell wall-binding repeat-containing protein
866,268	A→T	100%					S51T (TCA→ACA)	JMW53_03745 ←	cell wall-binding repeat-containing protein
866,272	C→T	100%					G49G (GGG→GGA)	JMW53_03745 ←	cell wall-binding repeat-containing protein
866,287	T→C	100%					K44K (AAA→AAG)	JMW53_03745 ←	cell wall-binding repeat-containing protein
866,293	T→C	100%					A42A (GCA→GCG)	JMW53_03745 ←	cell wall-binding repeat-containing protein
866,326	G→A	100%					D31D (GAC→GAT)	JMW53_03745 ←	cell wall-binding repeat-containing protein
866,337	A→C	100%					S28A (TCT→GCT)	JMW53_03745 ←	cell wall-binding repeat-containing protein
866,427	A→G	100%					intergenic (-9/+24)	JMW53_03745 ← / ← JMW53_03750	cell wall-binding repeat-containing protein/PIG-L family deacetylase
866,445	A→G	100%					intergenic (-27/+6)	JMW53_03745 ← / ← JMW53_03750	cell wall-binding repeat-containing protein/PIG-L family deacetylase
866,544	A→C	100%					P205P (CCT→CCG)	JMW53_03750 ←	PIG-L family deacetylase
866,554	C→T	100%					R202K (AGA→AAA)	JMW53_03750 ←	PIG-L family deacetylase
866,589	T→C	100%					E190E (GAA→GAG)	JMW53_03750 ←	PIG-L family deacetylase
866,661	T→C	100%					E166E (GAA→GAG)	JMW53_03750 ←	PIG-L family deacetylase
866,691	T→G	100%					G156G (GGA→GGC)	JMW53_03750 ←	PIG-L family deacetylase
866,706	C→T	100%					E151E (GAG→GAA)	JMW53_03750 ←	PIG-L family deacetylase
866,748	T→A	100%					G137G (GGA→GGT)	JMW53_03750 ←	PIG-L family deacetylase
866,757	T→C	100%					K134K (AAA→AAG)	JMW53_03750 ←	PIG-L family deacetylase
866,763	C→T	100%					P132P (CCG→CCA)	JMW53_03750 ←	PIG-L family deacetylase
866,772	C→A	100%					T129T (ACG→ACT)	JMW53_03750 ←	PIG-L family deacetylase
866,775	T→C	100%					K128K (AAA→AAG)	JMW53_03750 ←	PIG-L family deacetylase
866,781	T→C	100%					K126K (AAA→AAG)	JMW53_03750 ←	PIG-L family deacetylase
866,789	C→T	100%					D124N (GAT→AAT)	JMW53_03750 ←	PIG-L family deacetylase
866,801	C→T	100%					E120K (GAA→AAA)	JMW53_03750 ←	PIG-L family deacetylase
866,917	T→C	100%					K81R (AAG→AGG)	JMW53_03750 ←	PIG-L family deacetylase
867,048	A→G	100%					C37C (TGT→TGC)	JMW53_03750 ←	PIG-L family deacetylase
867,210	G→A	100%					intergenic (-52/+55)	JMW53_03750 ← / ← JMW53_03755	PIG-L family deacetylase/cell wall-binding protein Cwp2
867,233	2 bp→CA	100%					intergenic (-75/+31)	JMW53_03750 ← / ← JMW53_03755	PIG-L family deacetylase/cell wall-binding protein Cwp2
867,337	T→G	100%					A600A (GCA→GCC)	JMW53_03755 ←	cell wall-binding protein Cwp2
867,412	A→T	100%					G575G (GGT→GGA)	JMW53_03755 ←	cell wall-binding protein Cwp2
867,456	G→A	100%					L561L (CTA→TTA)	JMW53_03755 ←	cell wall-binding protein Cwp2
867,508	T→A	100%					G543G (GGA→GGT)	JMW53_03755 ←	cell wall-binding protein Cwp2
867,646	G→T	100%					G497G (GGC→GGA)	JMW53_03755 ←	cell wall-binding protein Cwp2
867,669	G→A	100%					L490L (CTG→TTG)	JMW53_03755 ←	cell wall-binding protein Cwp2
867,684	A→C	100%					S485A (TCA→GCA)	JMW53_03755 ←	cell wall-binding protein Cwp2
867,766	C→T	100%					G457G (GGG→GGA)	JMW53_03755 ←	cell wall-binding protein Cwp2
867,775	A→G	100%					G454G (GGT→GGC)	JMW53_03755 ←	cell wall-binding protein Cwp2
867,802	C→A	100%					G445G (GGG→GGT)	JMW53_03755 ←	cell wall-binding protein Cwp2
867,817	T→C	100%					V440V (GTA→GTG)	JMW53_03755 ←	cell wall-binding protein Cwp2
867,846	A→C	100%					Y431D (TAT→GAT)	JMW53_03755 ←	cell wall-binding protein Cwp2
867,885	2 bp→GC	100%					coding (1251-1252/1872 nt)	JMW53_03755 ←	cell wall-binding protein Cwp2
867,913	T→C	100%					S408S (TCA→TCG)	JMW53_03755 ←	cell wall-binding protein Cwp2
867,916	C→T	100%					E407E (GAG→GAA)	JMW53_03755 ←	cell wall-binding protein Cwp2
867,919	T→C	100%					G406G (GGA→GGG)	JMW53_03755 ←	cell wall-binding protein Cwp2
867,949	G→A	100%					S396S (AGC→AGT)	JMW53_03755 ←	cell wall-binding protein Cwp2
867,985	T→C	100%					V384V (GTA→GTG)	JMW53_03755 ←	cell wall-binding protein Cwp2
868,015	C→T	100%					L374L (TTG→TTA)	JMW53_03755 ←	cell wall-binding protein Cwp2
868,042	G→A	100%					L365L (CTC→CTT)	JMW53_03755 ←	cell wall-binding protein Cwp2
868,129	C→T	100%					V336V (GTG→GTA)	JMW53_03755 ←	cell wall-binding protein Cwp2
868,174	T→A	100%					S321S (TCA→TCT)	JMW53_03755 ←	cell wall-binding protein Cwp2
868,180	A→T	100%					G319G (GGT→GGA)	JMW53_03755 ←	cell wall-binding protein Cwp2
868,225	G→A	100%					S304S (AGC→AGT)	JMW53_03755 ←	cell wall-binding protein Cwp2
868,336	T→A	100%					T267T (ACA→ACT)	JMW53_03755 ←	cell wall-binding protein Cwp2
868,346	A→G	100%					V264A (GTA→GCA)	JMW53_03755 ←	cell wall-binding protein Cwp2
868,441	T→C	100%					L232L (TTA→TTG)	JMW53_03755 ←	cell wall-binding protein Cwp2
868,455	C→T	100%					D228N (GAT→AAT)	JMW53_03755 ←	cell wall-binding protein Cwp2
868,459	A→G	100%					N226N (AAT→AAC)	JMW53_03755 ←	cell wall-binding protein Cwp2
868,483	T→C	100%					K218K (AAA→AAG)	JMW53_03755 ←	cell wall-binding protein Cwp2
868,488	T→C	100%					N217D (AAT→GAT)	JMW53_03755 ←	cell wall-binding protein Cwp2
868,548	T→C	100%					I197V (ATT→GTT)	JMW53_03755 ←	cell wall-binding protein Cwp2
868,558	T→A	100%					T193T (ACA→ACT)	JMW53_03755 ←	cell wall-binding protein Cwp2
868,569	C→T	100%					V190I (GTT→ATT)	JMW53_03755 ←	cell wall-binding protein Cwp2
868,590	2 bp→TT	100%					coding (546-547/1872 nt)	JMW53_03755 ←	cell wall-binding protein Cwp2
868,627	T→A	100%					R170S (AGA→AGT)	JMW53_03755 ←	cell wall-binding protein Cwp2
868,656	C→T	100%					A161T (GCT→ACT)	JMW53_03755 ←	cell wall-binding protein Cwp2
868,695	T→G	100%					I148L (ATT→CTT)	JMW53_03755 ←	cell wall-binding protein Cwp2
868,738	T→C	100%					E133E (GAA→GAG)	JMW53_03755 ←	cell wall-binding protein Cwp2
868,743	2 bp→AT	100%					coding (393-394/1872 nt)	JMW53_03755 ←	cell wall-binding protein Cwp2
868,750	G→T	100%					D129E (GAC→GAA)	JMW53_03755 ←	cell wall-binding protein Cwp2
868,816	C→A	100%					G107G (GGG→GGT)	JMW53_03755 ←	cell wall-binding protein Cwp2
868,858	C→T	100%					L93L (TTG→TTA)	JMW53_03755 ←	cell wall-binding protein Cwp2
868,902	C→T	100%					V79I (GTA→ATA)	JMW53_03755 ←	cell wall-binding protein Cwp2
868,919	T→G	100%					N73T (AAT→ACT)	JMW53_03755 ←	cell wall-binding protein Cwp2
868,972	A→G	100%					Y55Y (TAT→TAC)	JMW53_03755 ←	cell wall-binding protein Cwp2
868,987	T→C	100%					L50L (TTA→TTG)	JMW53_03755 ←	cell wall-binding protein Cwp2
869,002	T→C	100%					S45S (TCA→TCG)	JMW53_03755 ←	cell wall-binding protein Cwp2
869,010	G→A	100%					L43L (CTA→TTA)	JMW53_03755 ←	cell wall-binding protein Cwp2
869,050	C→T	100%					Q29Q (CAG→CAA)	JMW53_03755 ←	cell wall-binding protein Cwp2
869,076	G→T	100%					P21T (CCA→ACA)	JMW53_03755 ←	cell wall-binding protein Cwp2
869,103	T→C	100%					T12A (ACT→GCT)	JMW53_03755 ←	cell wall-binding protein Cwp2
869,156	A→T	100%					intergenic (-20/+394)	JMW53_03755 ← / ← secA	cell wall-binding protein Cwp2/preprotein translocase subunit SecA
869,259	T→C	100%					intergenic (-123/+291)	JMW53_03755 ← / ← secA	cell wall-binding protein Cwp2/preprotein translocase subunit SecA
869,313	C→T	100%					intergenic (-177/+237)	JMW53_03755 ← / ← secA	cell wall-binding protein Cwp2/preprotein translocase subunit SecA
869,361	T→A	100%					intergenic (-225/+189)	JMW53_03755 ← / ← secA	cell wall-binding protein Cwp2/preprotein translocase subunit SecA
869,381	G→A	100%					intergenic (-245/+169)	JMW53_03755 ← / ← secA	cell wall-binding protein Cwp2/preprotein translocase subunit SecA
869,477	T→G	100%					intergenic (-341/+73)	JMW53_03755 ← / ← secA	cell wall-binding protein Cwp2/preprotein translocase subunit SecA
869,498	T→C	100%					intergenic (-362/+52)	JMW53_03755 ← / ← secA	cell wall-binding protein Cwp2/preprotein translocase subunit SecA
869,539	T→C	100%					intergenic (-403/+11)	JMW53_03755 ← / ← secA	cell wall-binding protein Cwp2/preprotein translocase subunit SecA
869,543	C→A	100%					intergenic (-407/+7)	JMW53_03755 ← / ← secA	cell wall-binding protein Cwp2/preprotein translocase subunit SecA
869,574	C→T	100%					V774V (GTG→GTA)	secA ←	preprotein translocase subunit SecA
869,616	G→A	100%					D760D (GAC→GAT)	secA ←	preprotein translocase subunit SecA
869,646	C→T	100%					P750P (CCG→CCA)	secA ←	preprotein translocase subunit SecA
869,679	A→G	100%					Y739Y (TAT→TAC)	secA ←	preprotein translocase subunit SecA
869,751	C→T	100%					K715K (AAG→AAA)	secA ←	preprotein translocase subunit SecA
869,757	C→T	100%					L713L (TTG→TTA)	secA ←	preprotein translocase subunit SecA
869,765	A→C	100%					S711A (TCA→GCA)	secA ←	preprotein translocase subunit SecA
869,783	G→T	100%					L705I (CTT→ATT)	secA ←	preprotein translocase subunit SecA
869,796	T→C	100%					L700L (TTA→TTG)	secA ←	preprotein translocase subunit SecA
869,799	A→G	100%					D699D (GAT→GAC)	secA ←	preprotein translocase subunit SecA
869,805	A→T	100%					V697V (GTT→GTA)	secA ←	preprotein translocase subunit SecA
869,814	T→A	100%					S694S (TCA→TCT)	secA ←	preprotein translocase subunit SecA
869,826	A→G	100%					T690T (ACT→ACC)	secA ←	preprotein translocase subunit SecA
869,837	C→T	100%					V687I (GTT→ATT)	secA ←	preprotein translocase subunit SecA
869,841	T→C	100%					E685E (GAA→GAG)	secA ←	preprotein translocase subunit SecA
869,862	T→C	100%					V678V (GTA→GTG)	secA ←	preprotein translocase subunit SecA
869,865	T→A	100%					G677G (GGA→GGT)	secA ←	preprotein translocase subunit SecA
869,876	A→G	100%					L674L (TTA→CTA)	secA ←	preprotein translocase subunit SecA
869,879	G→A	100%					L673L (CTA→TTA)	secA ←	preprotein translocase subunit SecA
869,912	A→G	100%					Y662H (TAT→CAT)	secA ←	preprotein translocase subunit SecA
869,916	A→G	100%					F660F (TTT→TTC)	secA ←	preprotein translocase subunit SecA
869,948	C→T	100%					V650I (GTT→ATT)	secA ←	preprotein translocase subunit SecA
869,956	T→G	100%					N647T (AAT→ACT)	secA ←	preprotein translocase subunit SecA
869,982	T→C	100%					K638K (AAA→AAG)	secA ←	preprotein translocase subunit SecA
870,000	A→G	100%					D632D (GAT→GAC)	secA ←	preprotein translocase subunit SecA
870,009	A→T	100%					I629I (ATT→ATA)	secA ←	preprotein translocase subunit SecA
870,012	G→A	100%					D628D (GAC→GAT)	secA ←	preprotein translocase subunit SecA
870,015	T→C	100%					E627E (GAA→GAG)	secA ←	preprotein translocase subunit SecA
870,020	T→C	100%					N626D (AAT→GAT)	secA ←	preprotein translocase subunit SecA
870,027	A→T	100%					V623V (GTT→GTA)	secA ←	preprotein translocase subunit SecA
870,033	A→G	100%					N621N (AAT→AAC)	secA ←	preprotein translocase subunit SecA
870,135	A→T	100%					A587A (GCT→GCA)	secA ←	preprotein translocase subunit SecA
870,177	A→T	100%					T573T (ACT→ACA)	secA ←	preprotein translocase subunit SecA
870,213	C→T	100%					E561E (GAG→GAA)	secA ←	preprotein translocase subunit SecA
870,297	A→C	100%					G533G (GGT→GGG)	secA ←	preprotein translocase subunit SecA
870,306	T→C	100%					G530G (GGA→GGG)	secA ←	preprotein translocase subunit SecA
870,339	G→A	100%					H519H (CAC→CAT)	secA ←	preprotein translocase subunit SecA
870,377	T→C	100%					N507D (AAT→GAT)	secA ←	preprotein translocase subunit SecA
870,386	G→C	100%					Q504E (CAA→GAA)	secA ←	preprotein translocase subunit SecA
870,400	C→T	100%					R499K (AGA→AAA)	secA ←	preprotein translocase subunit SecA
870,426	C→T	100%					T490T (ACG→ACA)	secA ←	preprotein translocase subunit SecA
870,429	T→A	100%					G489G (GGA→GGT)	secA ←	preprotein translocase subunit SecA
870,468	T→C	100%					L476L (TTA→TTG)	secA ←	preprotein translocase subunit SecA
870,495	C→T	100%					A467A (GCG→GCA)	secA ←	preprotein translocase subunit SecA
870,559	C→T	100%					R446K (AGA→AAA)	secA ←	preprotein translocase subunit SecA
870,576	T→C	100%					K440K (AAA→AAG)	secA ←	preprotein translocase subunit SecA
870,579	T→C	100%					E439E (GAA→GAG)	secA ←	preprotein translocase subunit SecA
870,597	T→C	100%					V433V (GTA→GTG)	secA ←	preprotein translocase subunit SecA
870,609	T→C	100%					Q429Q (CAA→CAG)	secA ←	preprotein translocase subunit SecA
870,627	C→T	100%					R423R (AGG→AGA)	secA ←	preprotein translocase subunit SecA
870,652	T→C	100%					N415S (AAT→AGT)	secA ←	preprotein translocase subunit SecA
870,672	C→T	100%					K408K (AAG→AAA)	secA ←	preprotein translocase subunit SecA
870,690	T→C	100%					L402L (TTA→TTG)	secA ←	preprotein translocase subunit SecA
870,705	C→A	100%					V397V (GTG→GTT)	secA ←	preprotein translocase subunit SecA
870,804	T→G	100%					E364D (GAA→GAC)	secA ←	preprotein translocase subunit SecA
870,812	G→A	100%					L362L (CTA→TTA)	secA ←	preprotein translocase subunit SecA
870,816	G→A	100%					F360F (TTC→TTT)	secA ←	preprotein translocase subunit SecA
870,834	C→A	100%					V354V (GTG→GTT)	secA ←	preprotein translocase subunit SecA
870,861	A→T	100%					N345K (AAT→AAA)	secA ←	preprotein translocase subunit SecA
870,882	T→G	100%					A338A (GCA→GCC)	secA ←	preprotein translocase subunit SecA
870,975	G→A	100%					I307I (ATC→ATT)	secA ←	preprotein translocase subunit SecA
871,005	C→T	100%					K297K (AAG→AAA)	secA ←	preprotein translocase subunit SecA
871,008	A→G	100%					H296H (CAT→CAC)	secA ←	preprotein translocase subunit SecA
871,032	G→A	100%					H288H (CAC→CAT)	secA ←	preprotein translocase subunit SecA
871,050	A→G	100%					N282N (AAT→AAC)	secA ←	preprotein translocase subunit SecA
871,098	G→A	100%					S266S (AGC→AGT)	secA ←	preprotein translocase subunit SecA
871,140	A→G	100%					D252D (GAT→GAC)	secA ←	preprotein translocase subunit SecA
871,166	T→C	100%					I244V (ATT→GTT)	secA ←	preprotein translocase subunit SecA
871,175	T→C	100%					I241V (ATA→GTA)	secA ←	preprotein translocase subunit SecA
871,188	C→T	100%					L236L (TTG→TTA)	secA ←	preprotein translocase subunit SecA
871,191	T→C	100%					E235E (GAA→GAG)	secA ←	preprotein translocase subunit SecA
871,215	T→A	100%					G227G (GGA→GGT)	secA ←	preprotein translocase subunit SecA
871,236	C→T	100%					P220P (CCG→CCA)	secA ←	preprotein translocase subunit SecA
871,239	A→G	100%					T219T (ACT→ACC)	secA ←	preprotein translocase subunit SecA
871,269	T→A	100%					V209V (GTA→GTT)	secA ←	preprotein translocase subunit SecA
871,272	C→T	100%					E208E (GAG→GAA)	secA ←	preprotein translocase subunit SecA
871,284	G→A	100%					A204A (GCC→GCT)	secA ←	preprotein translocase subunit SecA
871,290	G→A	100%					N202N (AAC→AAT)	secA ←	preprotein translocase subunit SecA
871,308	C→T	100%					K196K (AAG→AAA)	secA ←	preprotein translocase subunit SecA
871,326	A→C	100%					V190V (GTT→GTG)	secA ←	preprotein translocase subunit SecA
871,347	A→G	100%					D183D (GAT→GAC)	secA ←	preprotein translocase subunit SecA
871,371	A→T	100%					G175G (GGT→GGA)	secA ←	preprotein translocase subunit SecA
871,395	C→T	100%					Q167Q (CAG→CAA)	secA ←	preprotein translocase subunit SecA
871,400	G→C	100%					Q166E (CAA→GAA)	secA ←	preprotein translocase subunit SecA
871,411	T→C	100%					N162S (AAT→AGT)	secA ←	preprotein translocase subunit SecA
871,414	T→G	100%					Q161P (CAA→CCA)	secA ←	preprotein translocase subunit SecA
871,427	C→A	100%					A157S (GCT→TCT)	secA ←	preprotein translocase subunit SecA
871,450	C→T	100%					G149D (GGT→GAT)	secA ←	preprotein translocase subunit SecA
871,470	T→A	100%					R142R (AGA→CGT)	secA ←	preprotein translocase subunit SecA
871,472	T→G	100%					R142R (AGA→CGT)	secA ←	preprotein translocase subunit SecA
871,479	T→C	100%					E139E (GAA→GAG)	secA ←	preprotein translocase subunit SecA
871,482	T→C	100%					K138K (AAA→AAG)	secA ←	preprotein translocase subunit SecA
871,500	G→A	100%					Y132Y (TAC→TAT)	secA ←	preprotein translocase subunit SecA
871,503	A→G	100%					D131D (GAT→GAC)	secA ←	preprotein translocase subunit SecA
871,542	A→G	100%					A118A (GCT→GCC)	secA ←	preprotein translocase subunit SecA
871,550	G→A	100%					L116L (CTA→TTA)	secA ←	preprotein translocase subunit SecA
871,574	2 bp→GA	100%					coding (321-322/2346 nt)	secA ←	preprotein translocase subunit SecA
871,590	T→A	100%					T102T (ACA→ACT)	secA ←	preprotein translocase subunit SecA
871,602	A→T	100%					A98A (GCT→GCA)	secA ←	preprotein translocase subunit SecA
871,605	G→T	100%					I97I (ATC→ATA)	secA ←	preprotein translocase subunit SecA
871,611	A→T	100%					G95G (GGT→GGA)	secA ←	preprotein translocase subunit SecA
871,629	C→A	100%					G89G (GGG→GGT)	secA ←	preprotein translocase subunit SecA
871,641	C→T	100%					Q85Q (CAG→CAA)	secA ←	preprotein translocase subunit SecA
871,653	T→C	100%					Q81Q (CAA→CAG)	secA ←	preprotein translocase subunit SecA
872,680	A→T	100%					L532L (CTT→CTA)	slpA ←	S-layer protein SlpA
872,682	2 bp→AA	100%					coding (1593-1594/2181 nt)	slpA ←	S-layer protein SlpA
872,695	T→A	100%					V527V (GTA→GTT)	slpA ←	S-layer protein SlpA
872,698	T→A	100%					V526V (GTA→GTT)	slpA ←	S-layer protein SlpA
872,701	G→A	100%					F525F (TTC→TTT)	slpA ←	S-layer protein SlpA
872,704	T→A	100%					A524A (GCA→GCT)	slpA ←	S-layer protein SlpA
872,715	C→T	100%					D521N (GAT→AAT)	slpA ←	S-layer protein SlpA
872,737	T→A	100%					I513I (ATA→ATT)	slpA ←	S-layer protein SlpA
872,752	A→T	100%					A508A (GCT→GCA)	slpA ←	S-layer protein SlpA
872,767	T→A	100%					G503G (GGA→GGT)	slpA ←	S-layer protein SlpA
872,770	T→A	100%					S502A (TCA→GCT)	slpA ←	S-layer protein SlpA
872,772	A→C	100%					S502A (TCA→GCT)	slpA ←	S-layer protein SlpA
872,779	T→A	100%					T499T (ACA→ACT)	slpA ←	S-layer protein SlpA
872,782	T→A	100%					V498V (GTA→GTT)	slpA ←	S-layer protein SlpA
872,818	T→A	100%					E486D (GAA→GAT)	slpA ←	S-layer protein SlpA
872,821	T→C	100%					K485K (AAA→AAG)	slpA ←	S-layer protein SlpA
872,836	A→T	100%					V480V (GTT→GTA)	slpA ←	S-layer protein SlpA
872,839	T→A	100%					G479G (GGA→GGT)	slpA ←	S-layer protein SlpA
872,850	G→A	100%					L476L (CTA→TTA)	slpA ←	S-layer protein SlpA
872,860	C→T	100%					K472K (AAG→AAA)	slpA ←	S-layer protein SlpA
873,728	C→T	?	100%				G183E (GGA→GAA)	slpA ←	S-layer protein SlpA
873,831	T→C	?		100%			K149E (AAA→GAA)	slpA ←	S-layer protein SlpA
874,260	A→T	100%					L6I (TTA→ATA)	slpA ←	S-layer protein SlpA
874,410	$(T)_{7\to6}$	100%					intergenic (-135/+78)	slpA ← / ← JMW53_03770	S-layer protein SlpA/cell wall-binding repeat-containing protein
874,431	2 bp→TC	100%					intergenic (-156/+56)	slpA ← / ← JMW53_03770	S-layer protein SlpA/cell wall-binding repeat-containing protein
874,434	T→A	100%					intergenic (-159/+54)	slpA ← / ← JMW53_03770	S-layer protein SlpA/cell wall-binding repeat-containing protein
874,452	T→C	100%					intergenic (-177/+36)	slpA ← / ← JMW53_03770	S-layer protein SlpA/cell wall-binding repeat-containing protein
874,532	A→G	100%					S516P (TCT→CCT)	JMW53_03770 ←	cell wall-binding repeat-containing protein
874,544	G→A	100%					L512L (CTG→TTG)	JMW53_03770 ←	cell wall-binding repeat-containing protein

874,599	C→T	100%					S493S (TCG→TCA)	JMW53_03770 ←	cell wall-binding repeat-containing protein
874,617	A→G	100%					F487F (TTT→TTC)	JMW53_03770 ←	cell wall-binding repeat-containing protein
874,647	C→T	100%					K477K (AAG→AAA)	JMW53_03770 ←	cell wall-binding repeat-containing protein
874,653	C→T	100%					T475T (ACG→ACA)	JMW53_03770 ←	cell wall-binding repeat-containing protein
874,662	A→G	100%					S472S (AGT→AGC)	JMW53_03770 ←	cell wall-binding repeat-containing protein
874,665	T→C	100%					K471K (AAA→AAG)	JMW53_03770 ←	cell wall-binding repeat-containing protein
874,695	C→T	100%					E461E (GAG→GAA)	JMW53_03770 ←	cell wall-binding repeat-containing protein
874,728	T→C	100%					E450E (GAA→GAG)	JMW53_03770 ←	cell wall-binding repeat-containing protein
874,767	G→A	100%					G437G (GGC→GGT)	JMW53_03770 ←	cell wall-binding repeat-containing protein
874,773	A→T	100%					D435E (GAT→GAA)	JMW53_03770 ←	cell wall-binding repeat-containing protein
874,820	T→C	100%					K420E (AAA→GAA)	JMW53_03770 ←	cell wall-binding repeat-containing protein
874,824	A→T	100%					V418V (GTT→GTA)	JMW53_03770 ←	cell wall-binding repeat-containing protein
874,836	T→C	100%					E414E (GAA→GAG)	JMW53_03770 ←	cell wall-binding repeat-containing protein
874,909	C→T	100%					S390N (AGT→AAT)	JMW53_03770 ←	cell wall-binding repeat-containing protein
874,947	T→C	100%					V377V (GTA→GTG)	JMW53_03770 ←	cell wall-binding repeat-containing protein
874,957	T→C	100%					N374S (AAT→AGT)	JMW53_03770 ←	cell wall-binding repeat-containing protein
874,959	T→C	100%					I373M (ATA→ATG)	JMW53_03770 ←	cell wall-binding repeat-containing protein
875,007	G→A	100%					Y357Y (TAC→TAT)	JMW53_03770 ←	cell wall-binding repeat-containing protein
875,016	A→C	100%					V354V (GTT→GTG)	JMW53_03770 ←	cell wall-binding repeat-containing protein
875,082	T→C	100%					L332V (TTA→GTG)	JMW53_03770 ←	cell wall-binding repeat-containing protein
875,084	A→C	100%					L332V (TTA→GTG)	JMW53_03770 ←	cell wall-binding repeat-containing protein
875,091	A→C	100%					L329L (CTT→CTG)	JMW53_03770 ←	cell wall-binding repeat-containing protein
875,115	C→T	100%					G321G (GGG→GGA)	JMW53_03770 ←	cell wall-binding repeat-containing protein
875,128	T→C	100%					N317S (AAT→AGT)	JMW53_03770 ←	cell wall-binding repeat-containing protein
875,154	T→A	100%					A308A (GCA→GCT)	JMW53_03770 ←	cell wall-binding repeat-containing protein
875,163	A→G	100%					D305D (GAT→GAC)	JMW53_03770 ←	cell wall-binding repeat-containing protein
875,166	C→T	100%					L304L (CTG→TTA)	JMW53_03770 ←	cell wall-binding repeat-containing protein
875,168	G→A	100%					L304L (CTG→TTA)	JMW53_03770 ←	cell wall-binding repeat-containing protein
875,219	A→G	100%					L287L (TTA→CTA)	JMW53_03770 ←	cell wall-binding repeat-containing protein
875,226	G→A	100%					I284I (ATC→ATT)	JMW53_03770 ←	cell wall-binding repeat-containing protein
875,229	T→C	100%					K283K (AAA→AAG)	JMW53_03770 ←	cell wall-binding repeat-containing protein
875,247	C→A	100%					V277V (GTG→GTT)	JMW53_03770 ←	cell wall-binding repeat-containing protein
875,312	C→A	100%					A256S (GCT→TCT)	JMW53_03770 ←	cell wall-binding repeat-containing protein
875,331	G→A	100%					Y249Y (TAC→TAT)	JMW53_03770 ←	cell wall-binding repeat-containing protein
875,397	C→T	100%					E227E (GAG→GAA)	JMW53_03770 ←	cell wall-binding repeat-containing protein
875,475	C→A	100%					L201L (CTG→CTT)	JMW53_03770 ←	cell wall-binding repeat-containing protein
875,478	A→C	100%					A200A (GCT→GCG)	JMW53_03770 ←	cell wall-binding repeat-containing protein
875,490	C→T	100%					V196I (GTG→ATA)	JMW53_03770 ←	cell wall-binding repeat-containing protein
875,492	C→T	100%					V196I (GTG→ATA)	JMW53_03770 ←	cell wall-binding repeat-containing protein
875,516	C→T	100%					V188I (GTT→ATT)	JMW53_03770 ←	cell wall-binding repeat-containing protein
875,518	T→C	100%					N187S (AAT→AGT)	JMW53_03770 ←	cell wall-binding repeat-containing protein
875,544	T→C	100%					A178A (GCA→GCG)	JMW53_03770 ←	cell wall-binding repeat-containing protein
875,571	C→T	100%					A169A (GCG→GCA)	JMW53_03770 ←	cell wall-binding repeat-containing protein
875,624	T→C	100%					N152D (AAT→GAT)	JMW53_03770 ←	cell wall-binding repeat-containing protein
875,631	C→T	100%					K149K (AAG→AAA)	JMW53_03770 ←	cell wall-binding repeat-containing protein
875,646	C→T	100%					E144E (GAG→GAA)	JMW53_03770 ←	cell wall-binding repeat-containing protein
875,673	T→A	100%					I135V (ATA→GTT)	JMW53_03770 ←	cell wall-binding repeat-containing protein
875,675	T→C	100%					I135V (ATA→GTT)	JMW53_03770 ←	cell wall-binding repeat-containing protein
875,684	2 bp→TT	100%					coding (393-394/1590 nt)	JMW53_03770 ←	cell wall-binding repeat-containing protein
875,760	T→C	100%					G106G (GGA→GGG)	JMW53_03770 ←	cell wall-binding repeat-containing protein
875,773	G→A	100%					A102V (GCT→GTT)	JMW53_03770 ←	cell wall-binding repeat-containing protein
875,805	G→A	100%					D91D (GAC→GAT)	JMW53_03770 ←	cell wall-binding repeat-containing protein
875,810	G→A	100%					P90S (CCA→TCA)	JMW53_03770 ←	cell wall-binding repeat-containing protein
875,824	C→T	100%					R85K (AGA→AAA)	JMW53_03770 ←	cell wall-binding repeat-containing protein
875,883	G→A	100%					D65D (GAC→GAT)	JMW53_03770 ←	cell wall-binding repeat-containing protein
875,891	G→A	100%					L63L (CTA→TTA)	JMW53_03770 ←	cell wall-binding repeat-containing protein
875,928	C→T	100%					T50T (ACG→ACA)	JMW53_03770 ←	cell wall-binding repeat-containing protein
875,955	C→T	100%					T41T (ACG→ACA)	JMW53_03770 ←	cell wall-binding repeat-containing protein
875,991	A→G	100%					S29S (AGT→AGC)	JMW53_03770 ←	cell wall-binding repeat-containing protein
876,014	G→T	100%					P22T (CCA→ACA)	JMW53_03770 ←	cell wall-binding repeat-containing protein
876,078	C→A	100%					intergenic (-1/+217)	JMW53_03770 ← / ← JMW53_03775	cell wall-binding repeat-containing protein/cell wall-binding protein Cwp11
876,156	C→T	100%					intergenic (-79/+139)	JMW53_03770 ← / ← JMW53_03775	cell wall-binding repeat-containing protein/cell wall-binding protein Cwp11
876,178	T→C	100%					intergenic (-101/+117)	JMW53_03770 ← / ← JMW53_03775	cell wall-binding repeat-containing protein/cell wall-binding protein Cwp11
876,188	C→T	100%					intergenic (-111/+107)	JMW53_03770 ← / ← JMW53_03775	cell wall-binding repeat-containing protein/cell wall-binding protein Cwp11
876,202	C→A	100%					intergenic (-125/+93)	JMW53_03770 ← / ← JMW53_03775	cell wall-binding repeat-containing protein/cell wall-binding protein Cwp11
876,245	G→C	100%					intergenic (-168/+50)	JMW53_03770 ← / ← JMW53_03775	cell wall-binding repeat-containing protein/cell wall-binding protein Cwp11
876,268	A→C	100%					intergenic (-191/+27)	JMW53_03770 ← / ← JMW53_03775	cell wall-binding repeat-containing protein/cell wall-binding protein Cwp11
876,280	C→G	100%					intergenic (-203/+15)	JMW53_03770 ← / ← JMW53_03775	cell wall-binding repeat-containing protein/cell wall-binding protein Cwp11
876,301	G→A	100%					S532S (AGC→AGT)	JMW53_03775 ←	cell wall-binding protein Cwp11
876,307	T→C	100%					E530E (GAA→GAG)	JMW53_03775 ←	cell wall-binding protein Cwp11
876,347	C→T	100%					G517D (GGT→GAT)	JMW53_03775 ←	cell wall-binding protein Cwp11
876,355	T→C	100%					L514L (TTA→TTG)	JMW53_03775 ←	cell wall-binding protein Cwp11
876,392	T→C	100%					D502G (GAT→GGT)	JMW53_03775 ←	cell wall-binding protein Cwp11
876,433	C→A	100%					V488V (GTG→GTT)	JMW53_03775 ←	cell wall-binding protein Cwp11
876,436	C→T	100%					E487E (GAG→GAA)	JMW53_03775 ←	cell wall-binding protein Cwp11
876,475	T→C	100%					T474T (ACA→ACG)	JMW53_03775 ←	cell wall-binding protein Cwp11
876,484	C→G	100%					K471N (AAG→AAC)	JMW53_03775 ←	cell wall-binding protein Cwp11
876,526	T→C	100%					R457R (AGA→AGG)	JMW53_03775 ←	cell wall-binding protein Cwp11
876,547	T→C	100%					V450V (GTA→GTG)	JMW53_03775 ←	cell wall-binding protein Cwp11
876,565	A→T	100%					G444G (GGT→GGA)	JMW53_03775 ←	cell wall-binding protein Cwp11
894,734	T→C					100%	intergenic (-595/+52)	tnpB ← / ← JMW53_03860	IS200/IS605 family element transposase accessory protein TnpB/hydroxyacid dehydrogenase
977,737	$(T)_{8\to7}$					100%	coding (16/1014 nt)	JMW53_04180 ←	iron ABC transporter permease
992,738	$(GTGATATTACAGTTGATATAA)_{1\to2}$	100%					coding (248/1719 nt)	JMW53_04245 ←	putative 2-aminoethylphosphonate ABC transporter permease subunit
996,286	A→G			100%			I388T (ATA→ACA)	JMW53_04265 ←	V-type ATP synthase subunit B
1,006,264	C→T	100%					R52K (AGG→AAG)	JMW53_04310 ←	L,D-transpeptidase/peptidoglycan binding protein
1,022,245	299 bp→36 bp	100%					intergenic (-1855/+213)	JMW53_04370 ← / ← cas2	N-acetyltransferase/CRISPR-associated endonuclease Cas2
1,090,737	$(A)_{10\to9}$					100%	intergenic (-17/+194)	JMW53_04665 ← / ← JMW53_04670	CarD family transcriptional regulator/hypothetical protein
1,136,075	G→T	100%					intergenic (-88/+520)	JMW53_04850 ← / ← JMW53_04855	glycoside hydrolase family 31 protein/hypothetical protein
1,155,111	G→C	100%		100%		100%	I245M (ATC→ATG)	JMW53_04935 ←	glycoside hydrolase family 1 protein
1,164,412	G→T			100%			T351K (ACA→AAA)	JMW53_04965 ←	PTS transporter subunit EIIC
1,176,126	T→C	100%					Y43C (TAT→TGT)	JMW53_05005 ←	hypothetical protein
1,199,321	A→G	100%					S138S (TCT→TCC)	JMW53_05100 ←	ABC transporter ATP-binding protein
1,242,302	T→C		100%				G1044G (GGA→GGG)	JMW53_05270 ←	hypothetical protein
1,248,401	G→A	100%					N87N (AAC→AAT)	JMW53_05290 ←	hypothetical protein
1,252,710	$(A)_{8\to7}$					100%	intergenic (-587/-67)	smpB ← / → JMW53_05315	SsrA-binding protein SmpB/AIM24 family protein
1,299,409	A→G					100%	Y138C (TAC→TGC)	JMW53_05505 →	ABC transporter ATP-binding protein
1,305,895	A→G	100%					intergenic (-481/-125)	JMW53_05525 ← / → JMW53_05530	conserved phage C-terminal domain-containing protein/AraC family transcriptional regulator
1,318,756	C→T	100%					A144T (GCT→ACT)	JMW53_05580 ←	membrane protein insertase YidC
1,329,435	C→T	100%					L480L (CTG→CTA)	JMW53_05640 ←	excinuclease ABC subunit UvrA
1,359,538	A→G	100%					F491F (TTT→TTC)	prdA ←	D-proline reductase (dithiol) proprotein PrdA
1,360,561	Δ21 bp	100%					coding (430-450/1881 nt)	prdA ←	D-proline reductase (dithiol) proprotein PrdA
1,374,614	G→T					100%	intergenic (-342/+686)	JMW53_05830 ← / ← rgaR	S8 family serine peptidase/two-component system response regulator RgaR
1,375,595	G→A	100%	100%	100%	100%	100%	S139F (TCT→TTT)	rgaR ←	two-component system response regulator RgaR
1,375,893	C→A				100%		G40W (GGG→TGG)	rgaR ←	two-component system response regulator RgaR
1,376,008	C→T			100%			M1M (ATG→ATA) †	rgaR ←	two-component system response regulator RgaR
1,399,618	A→G					100%	I71T (ATA→ACA)	JMW53_05930 ←	SIS domain-containing protein
1,401,675	G→A	100%					Q82* (CAA→TAA)	JMW53_05945 ←	PTS mannose transporter subunit IIA
1,412,927	T→C	100%					R102G (AGA→GGA)	JMW53_05980 ←	glucose-6-phosphate isomerase
1,442,668	Δ6,059 bp	100%						[JMW53_06115]–[JMW53_06170]	[JMW53_06115], JMW53_06120, JMW53_06125, JMW53_06130, JMW53_06135, JMW53_06140, JMW53_06145, JMW53_06150, JMW53_06155, JMW53_06160, JMW53_06165, [JMW53_06170]
1,463,984	T→G	100%					T285T (ACA→ACC)	JMW53_06235 ←	spore surface glycoprotein BclB
1,522,621	A→G					100%	L21L (TTG→CTG)	JMW53_06465 ←	sugar ABC transporter permease
1,530,126	G→A	100%		100%		100%	P293L (CCT→CTT)	hemB ←	porphobilinogen synthase
1,555,174	Δ3 bp	100%					coding (882-884/1323 nt)	JMW53_06620 ←	PTS sugar transporter subunit IIC
1,559,832	C→T	100%					intergenic (-44/+285)	JMW53_06640 ← / ← gatY	BglG family transcription antiterminator/tagatose-bisphosphate aldolase subunit GatY
1,575,588	C→T	100%					V16I (GTT→ATT)	JMW53_06720 ←	hypothetical protein
1,606,632	A→C	100%					intergenic (-42/+78)	JMW53_06895 ← / ← spoVT	polysaccharide biosynthesis protein/stage V sporulation protein T
1,632,762	G→A	100%					H315H (CAC→CAT)	JMW53_07010 ←	M20/M25/M40 family metallo-hydrolase
1,643,993	A→G	100%					S2S (AGT→AGC)	metG ←	methionine--tRNA ligase
1,770,973	A→G	100%					F283S (TTT→TCT)	JMW53_07670 ←	PTS sugar transporter subunit IIC/EAL domain-containing protein
1,806,891	G→A				100%		D426N (GAT→AAT)	gyrB →	DNA topoisomerase (ATP-hydrolyzing) subunit B
1,807,521	$(AG)_{3\to4}$	100%					intergenic (+4/-14)	gyrB → / → gyrA	DNA topoisomerase (ATP-hydrolyzing) subunit B/DNA gyrase subunit A
1,807,779	C→T					100%	T82I (ACT→ATT)	gyrA →	DNA gyrase subunit A
1,840,728	A→G	100%					noncoding (60/77 nt)	JMW53_08230 →	tRNA-Arg
1,854,822	A→G	100%					intergenic (+63/-26)	disA → / → JMW53_08285	DNA integrity scanning protein DisA/PIN/TRAM domain-containing protein
1,865,620	C→T					100%	A88T (GCA→ACA)	JMW53_08320 ←	response regulator transcription factor
1,881,319	T→C	100%					V199A (GTT→GCT)	JMW53_08385 →	lipoate--protein ligase
1,990,787	C→T	100%					L49L (CTA→TTA)	JMW53_09080 →	transcription antiterminator
1,994,605	A→C	100%					I404L (ATT→CTT)	celB →	PTS cellobiose transporter subunit IIC
2,071,322	A→G		100%				intergenic (+140/-125)	JMW53_09435 → / → JMW53_09440	GGDEF domain-containing protein/PTS sugar transporter subunit IIA
2,071,606	A→G	100%					I54V (ATA→GTA)	JMW53_09440 →	PTS sugar transporter subunit IIA
2,073,535	C→G					100%	A21G (GCC→GGC)	JMW53_09445 →	PTS sugar transporter subunit IIA
2,087,928	A→C	100%		100%		100%	I388L (ATT→CTT)	JMW53_09520 →	phosphoribosylformylglycinamidine synthase
2,094,033	A→G	100%					K255E (AAA→GAA)	rfbB →	dTDP-glucose 4,6-dehydratase
2,121,648	G→T				100%		D260Y (GAT→TAT)	JMW53_09675 →	AraC family transcriptional regulator
2,122,976	C→T					100%	S44F (TCT→TTT)	JMW53_09690 →	pyridoxamine 5'-phosphate oxidase family protein
2,123,004	T→C					100%	L53L (CTT→CTC)	JMW53_09690 →	pyridoxamine 5'-phosphate oxidase family protein
2,133,383	+AATATA	100%					intergenic (+145/-172)	JMW53_09745 → / → JMW53_09750	M20 family metallopeptidase/DUF2185 domain-containing protein
2,134,314	T→C	100%					L45P (CTT→CCT)	JMW53_09755 →	helix-turn-helix transcriptional regulator
2,144,706	T→C	100%					D417D (GAT→GAC)	JMW53_09805 →	sugar ABC transporter ATP-binding protein
2,179,123	T→G	100%					intergenic (-778/-89)	JMW53_09945 ← / → adhE	putative manganese-dependent inorganic diphosphatase/bifunctional acetaldehyde-CoA/alcohol dehydrogenase
2,195,025	A→G	100%	100%	100%	100%	100%	I46M (ATA→ATG)	JMW53_10000 →	metallophosphoesterase
2,225,466	Δ106,711 bp	100%		100%		100%		[rlmD]–[JMW53_10690]	**113 genes** Show
2,277,601	Δ54,576 bp	Δ	100%	Δ		Δ		JMW53_10425–[JMW53_10690]	**54 genes** Show
2,353,803	T→A	100%					N473K (AAT→AAA)	JMW53_10790 →	DUF2075 domain-containing protein
2,369,242	T→C	100%					intergenic (+188/-59)	JMW53_10855 → / → JMW53_10860	helix-turn-helix transcriptional regulator/ATP-binding domain-containing protein
2,370,847	G→A	100%					G516D (GGC→GAC)	JMW53_10860 →	ATP-binding domain-containing protein
2,374,365	T→C	100%					D150D (GAT→GAC)	JMW53_10875 →	ABC transporter permease
2,398,743	C→T					100%	T12I (ACA→ATA)	JMW53_11000 →	hypothetical protein
2,417,470	C→T	100%					W697* (TGG→TAG)	JMW53_11110 ←	ABC transporter permease
2,463,608	A→G			100%			N197D (AAT→GAT)	JMW53_11290 →	diguanylate cyclase
2,486,502	$(GTAAAAGA)_{10\to7}$				100%		intergenic (+282/-269)	JMW53_11390 → / → glsA	nucleoside kinase/glutaminase A
2,543,771	A→G	100%					G112G (GGT→GGC)	JMW53_11620 ←	plasmid stabilization protein
2,552,082	T→G	100%		100%		100%	K92Q (AAA→CAA)	JMW53_11660 ←	TetR/AcrR family transcriptional regulator
2,564,439	G→A	100%					intergenic (-462/-69)	JMW53_11725 ← / → JMW53_11730	Crp/Fnr family transcriptional regulator/PadR family transcriptional regulator
2,567,945	A→C	100%					intergenic (+466/-248)	JMW53_11740 → / → JMW53_11745	radical SAM protein/GNAT family N-acetyltransferase
2,576,081	$(TGGATTAAGTA)_{2\to3}$	100%					intergenic (+156/-135)	JMW53_11785 → / → JMW53_11790	GntR family transcriptional regulator/S9 family peptidase
2,604,816	A→G	100%					R1901G (AGA→GGA)	tcdA →	glycosylating toxin TcdA
2,639,626	C→T	100%					G518D (GGT→GAT)	JMW53_12025 ←	peptidase M3
2,667,390	$(A)_{6\to5}$		100%				intergenic (+105/-149)	JMW53_12110 → / → JMW53_12115	arginine--tRNA ligase/hypothetical protein
2,674,518	G→A	100%					K255K (AAG→AAA)	JMW53_12145 →	bifunctional enoyl-CoA hydratase/phosphate acetyltransferase
2,675,891	G→A	100%					R207K (AGA→AAA)	cooS →	anaerobic carbon-monoxide dehydrogenase catalytic subunit
2,701,075	T→C	100%					intergenic (+264/+41)	JMW53_12265 → / ← JMW53_12270	hypothetical protein/alanine--glyoxylate aminotransferase family protein
2,728,558	T→C	100%					intergenic (+33/-58)	JMW53_12370 → / → JMW53_12375	calcium/sodium antiporter/DEAD/DEAH box helicase
2,761,323	$(A)_{8\to9}$	100%					intergenic (-105/-73)	JMW53_12535 ← / → nadE	sulfite exporter TauE/SafE family protein/NAD(+) synthase
2,789,162	A→G	100%					D209G (GAT→GGT)	JMW53_12660 →	ABC transporter permease
2,793,305	G→A		100%				intergenic (+243/-387)	JMW53_12685 → / → JMW53_12690	MBL fold metallo-hydrolase/transposase
2,808,374	G→A	100%					intergenic (+53/-46)	JMW53_12760 → / → JMW53_12765	fumarylacetoacetate hydrolase family protein/phosphoribosylaminoimidazolecarboxamide formyltransferase
2,939,936	A→G					100%	intergenic (+379/-3)	JMW53_13320 → / → JMW53_13325	CotH kinase family protein/hypothetical protein
2,956,791	T→A	?				100%	intergenic (+161/-165)	JMW53_13390 → / → JMW53_13395	16S ribosomal RNA/23S ribosomal RNA
2,994,652	G→A	100%					A202A (GCG→GCA)	JMW53_13520 →	3-hydroxybutyryl-CoA dehydrogenase
3,003,229	A→C	100%	100%	100%	100%	100%	noncoding (117/181 nt)	ssrS →	6S RNA
3,008,141	G→T			100%			M352I (ATG→ATT)	JMW53_13600 →	PLP-dependent aminotransferase family protein
3,055,294	A→G	100%	100%	100%	100%	100%	A174A (GCA→GCG)	JMW53_13830 →	RnfABCDGE type electron transport complex subunit D
3,063,665	G→A	100%					E369E (GAG→GAA)	JMW53_13880 →	penicillin-binding protein
3,064,735	C→T				100%		A726V (GCA→GTA)	JMW53_13880 →	penicillin-binding protein
3,086,122	T→C	100%					V10V (GTT→GTC)	JMW53_13995 →	electron transfer flavoprotein subunit beta/FixA family protein
3,095,358	C→T		100%				T3I (ACA→ATA)	JMW53_14040 →	ketoacyl-ACP synthase III
3,102,045	$(AAAATATTAT)_{2\to1}$			100%			coding (1183-1192/1719 nt)	JMW53_14070 →	GGDEF domain-containing protein
3,106,534	2 bp→AA	100%					coding (546-547/1314 nt)	JMW53_14090 →	amino acid permease
3,137,567	T→G	100%	100%	100%	100%	100%	I290R (ATA→AGA)	JMW53_14260 →	phosphopentomutase
3,150,379	38 bp→37 bp	100%					intergenic (+316/+638)	JMW53_14315 → / ← JMW53_14320	hypothetical protein/helix-turn-helix transcriptional regulator
3,197,456	G→T	100%					A214S (GCT→TCT)	nifS →	cysteine desulfurase NifS
3,206,597	A→T	100%					intergenic (+237/-45)	JMW53_14590 → / → JMW53_14595	DUF1292 domain-containing protein/transcriptional repressor
3,239,226	T→A	100%					N45K (AAT→AAA)	JMW53_14755 →	polysaccharide deacetylase family protein
3,246,299	G→A	100%					Q55Q (CAG→CAA)	mnmH →	tRNA 2-selenouridine(34) synthase MnmH
3,247,178	A→C	100%					G348G (GGA→GGC)	mnmH →	tRNA 2-selenouridine(34) synthase MnmH
3,277,482	A→G	100%					intergenic (+90/-44)	JMW53_14915 → / → JMW53_14920	lantibiotic immunity ABC transporter MutG family permease subunit/sensor histidine kinase
3,298,120	T→A				100%		F364Y (TTT→TAT)	JMW53_15030 →	chromosome condensation regulator
3,381,426	G→T	100%					intergenic (+217/-25)	JMW53_15375 → / → JMW53_15380	YitT family protein/hypothetical protein
3,382,017	A→C	100%		100%		100%	I189I (ATA→ATC)	JMW53_15380 →	hypothetical protein
3,398,089	G→A					100%	S356S (AGC→AGT)	JMW53_15475 ←	DUF1648 domain-containing protein
3,400,608	C→T	100%					P56S (CCA→TCA)	JMW53_15490 →	response regulator transcription factor
3,422,186	C→T				100%		intergenic (+163/-158)	JMW53_15550 → / → JMW53_15555	EAL domain-containing protein/ferrous iron transport protein A
3,428,522	T→C	100%					intergenic (+32/-2)	JMW53_15585 → / → JMW53_15590	ABC transporter ATP-binding protein/NrtA/SsuA/CpmA family ABC transporter substrate-binding protein
3,502,883	T→C	100%					F196F (TTT→TTC)	JMW53_15915 →	Fic family protein
3,505,844	T→G	100%					G71G (GGT→GGG)	JMW53_15935 →	hemolysin III family protein
3,559,560	C→T	100%					K294K (AAG→AAA)	JMW53_16185 ←	hypothetical protein
3,577,261	A→G	100%					T579A (ACT→GCT)	JMW53_16275 ←	CPBP family intramembrane metalloprotease
3,626,176	T→C	100%					intergenic (+395/-23)	gcvPB → / → JMW53_16470	aminomethyl-transferring glycine dehydrogenase subunit GcvPB/HAD-IC family P-type ATPase
3,633,412	101 bp→36 bp	100%					intergenic (-675/-1590)	JMW53_16495 ← / → JMW53_16500	amidohydrolase family protein/hypothetical protein
3,658,729	A→G	100%					E187G (GAA→GGA)	JMW53_16620 →	hypothetical protein
3,695,697	A→G	100%					V78A (GTT→GCT)	JMW53_16810 ←	DUF3795 domain-containing protein
3,766,073	A→G	100%					intergenic (+428/-7)	JMW53_17155 → / → JMW53_17160	arsenate reductase family protein/DUF4253 domain-containing protein
3,848,627	A→C	100%					P121P (CCA→CCC)	JMW53_17510 →	YtxH domain-containing protein
3,885,772	G→A	100%					intergenic (+317/-12)	JMW53_17715 → / → JMW53_17720	OmpA family protein/MBL fold metallo-hydrolase
3,904,177	G→A	100%					S144N (AGT→AAT)	JMW53_17810 →	YitT family protein
3,905,458	C→T	100%					intergenic (-53/+137)	JMW53_17815 ← / ← JMW53_17820	GNAT family N-acetyltransferase/LysR family transcriptional regulator
3,910,112	A→G					100%	I150T (ATC→ACC)	JMW53_17830 ←	FtsX-like permease family protein
3,926,229	C→A			100%			G95G (GGG→GGT)	JMW53_17910 ←	prolyl-tRNA synthetase associated domain-containing protein
3,960,160	+65 bp	100%					intergenic (+875/+3049)	JMW53_18090 → / ← JMW53_18095	helix-turn-helix transcriptional regulator/hypothetical protein
3,961,547	Δ66 bp	100%					intergenic (+2262/+1597)	JMW53_18090 → / ← JMW53_18095	helix-turn-helix transcriptional regulator/hypothetical protein
3,962,339	563 bp→36 bp	100%					intergenic (+3054/+308)	JMW53_18090 → / ← JMW53_18095	helix-turn-helix transcriptional regulator/hypothetical protein
3,978,745	Δ17,910 bp	100%		100%				JMW53_18190–[JMW53_18285]	**20 genes** Show
3,982,996	A→G	Δ				100%	Y555Y (TAT→TAC)	tet(M) ←	tetracycline resistance ribosomal protection protein Tet(M)
3,983,017	T→C	Δ				100%	Q548Q (CAA→CAG)	tet(M) ←	tetracycline resistance ribosomal protection protein Tet(M)
3,983,023	T→C	Δ				100%	A546A (GCA→GCG)	tet(M) ←	tetracycline resistance ribosomal protection protein Tet(M)
3,983,080	A→G	Δ				100%	V527V (GTT→GTC)	tet(M) ←	tetracycline resistance ribosomal protection protein Tet(M)
3,983,209	G→A	Δ				100%	G484G (GGC→GGT)	tet(M) ←	tetracycline resistance ribosomal protection protein Tet(M)
3,983,215	T→G	Δ				100%	R482R (CGA→CGC)	tet(M) ←	tetracycline resistance ribosomal protection protein Tet(M)
3,983,245	C→T	Δ				100%	S472S (TCG→TCA)	tet(M) ←	tetracycline resistance ribosomal protection protein Tet(M)
3,983,287	T→C	Δ				100%	V458M (GTA→ATG)	tet(M) ←	tetracycline resistance ribosomal protection protein Tet(M)
3,983,289	C→T	Δ				100%	V458M (GTA→ATG)	tet(M) ←	tetracycline resistance ribosomal protection protein Tet(M)
3,983,293	G→A	Δ				100%	S456S (AGC→AGT)	tet(M) ←	tetracycline resistance ribosomal protection protein Tet(M)
3,983,296	C→T	Δ				100%	G455G (GGG→GGA)	tet(M) ←	tetracycline resistance ribosomal protection protein Tet(M)
3,983,299	T→C	Δ				100%	L454L (TTA→TTG)	tet(M) ←	tetracycline resistance ribosomal protection protein Tet(M)
3,983,302	T→C	Δ				100%	P453P (CCA→CCG)	tet(M) ←	tetracycline resistance ribosomal protection protein Tet(M)
3,983,313	C→A	Δ				100%	A450S (GCA→TCA)	tet(M) ←	tetracycline resistance ribosomal protection protein Tet(M)
3,983,320	A→T	Δ				100%	L447L (CTT→TTA)	tet(M) ←	tetracycline resistance ribosomal protection protein Tet(M)
3,983,322	G→A	Δ				100%	L447L (CTT→TTA)	tet(M) ←	tetracycline resistance ribosomal protection protein Tet(M)
3,983,341	G→A	Δ				100%	P440P (CCC→CCT)	tet(M) ←	tetracycline resistance ribosomal protection protein Tet(M)
3,983,347	C→T	Δ				100%	P438P (CCG→CCA)	tet(M) ←	tetracycline resistance ribosomal protection protein Tet(M)
3,983,350	T→C	Δ				100%	P437P (CCA→CCG)	tet(M) ←	tetracycline resistance ribosomal protection protein Tet(M)
3,983,374	C→T	Δ				100%	E429E (GAG→GAA)	tet(M) ←	tetracycline resistance ribosomal protection protein Tet(M)
3,983,380	T→A	Δ				100%	K427N (AAA→AAT)	tet(M) ←	tetracycline resistance ribosomal protection protein Tet(M)
3,983,395	T→C	Δ				100%	E422E (GAA→GAG)	tet(M) ←	tetracycline resistance ribosomal protection protein Tet(M)
3,983,418	T→C	Δ				100%	K415E (AAG→GAG)	tet(M) ←	tetracycline resistance ribosomal protection protein Tet(M)
3,983,424	T→G	Δ				100%	I413L (ATA→CTA)	tet(M) ←	tetracycline resistance ribosomal protection protein Tet(M)
3,983,427	T→C	Δ				100%	K412E (AAA→GAA)	tet(M) ←	tetracycline resistance ribosomal protection protein Tet(M)
3,983,430	C→T	Δ				100%	V411I (GTA→ATA)	tet(M) ←	tetracycline resistance ribosomal protection protein Tet(M)
3,983,455	T→C	Δ				100%	L402L (CTA→CTG)	tet(M) ←	tetracycline resistance ribosomal protection protein Tet(M)
3,983,458	A→T	Δ				100%	A401A (GCT→GCA)	tet(M) ←	tetracycline resistance ribosomal protection protein Tet(M)
3,983,463	A→T	Δ				100%	C400S (TGT→AGT)	tet(M) ←	tetracycline resistance ribosomal protection protein Tet(M)
3,983,465	G→A	Δ				100%	T399I (ACT→ATT)	tet(M) ←	tetracycline resistance ribosomal protection protein Tet(M)
3,983,503	G→A	Δ				100%	I386I (ATC→ATT)	tet(M) ←	tetracycline resistance ribosomal protection protein Tet(M)
3,983,527	A→G	Δ				100%	Y378Y (TAT→TAC)	tet(M) ←	tetracycline resistance ribosomal protection protein Tet(M)
3,983,534	T→C	Δ				100%	Q376R (CAA→CGA)	tet(M) ←	tetracycline resistance ribosomal protection protein Tet(M)
3,983,545	G→A	Δ				100%	D372D (GAC→GAT)	tet(M) ←	tetracycline resistance ribosomal protection protein Tet(M)
3,983,551	G→A	Δ				100%	D370D (GAC→GAT)	tet(M) ←	tetracycline resistance ribosomal protection protein Tet(M)
3,983,554	G→T	Δ				100%	S369S (TCC→TCA)	tet(M) ←	tetracycline resistance ribosomal protection protein Tet(M)
3,983,563	T→C	Δ				100%	L366L (TTA→TTG)	tet(M) ←	tetracycline resistance ribosomal protection protein Tet(M)
3,983,569	T→G	Δ				100%	A364A (GCA→GCC)	tet(M) ←	tetracycline resistance ribosomal protection protein Tet(M)
3,983,578	T→C	Δ				100%	L361L (TTA→TTG)	tet(M) ←	tetracycline resistance ribosomal protection protein Tet(M)
3,983,587	C→T	Δ				100%	R358R (AGG→AGA)	tet(M) ←	tetracycline resistance ribosomal protection protein Tet(M)
3,983,590	T→C	Δ				100%	Q357Q (CAA→CAG)	tet(M) ←	tetracycline resistance ribosomal protection protein Tet(M)
3,983,595	G→C	Δ				100%	Q356E (CAA→GAA)	tet(M) ←	tetracycline resistance ribosomal protection protein Tet(M)
3,983,602	G→A	Δ				100%	S353S (AGC→AGT)	tet(M) ←	tetracycline resistance ribosomal protection protein Tet(M)
3,983,623	C→T	Δ				100%	L346L (CTG→CTA)	tet(M) ←	tetracycline resistance ribosomal protection protein Tet(M)
3,983,626	C→T	Δ				100%	L345L (CTG→CTA)	tet(M) ←	tetracycline resistance ribosomal protection protein Tet(M)
3,983,633	A→T	Δ				100%	L343H (CTC→CAC)	tet(M) ←	tetracycline resistance ribosomal protection protein Tet(M)
3,983,647	2 bp→CT	Δ				100%	coding (1013-1014/1920 nt)	tet(M) ←	tetracycline resistance ribosomal protection protein Tet(M)
3,983,650	C→T	Δ				100%	E337K (GAG→AAA)	tet(M) ←	tetracycline resistance ribosomal protection protein Tet(M)
3,983,652	C→T	Δ				100%	E337K (GAG→AAA)	tet(M) ←	tetracycline resistance ribosomal protection protein Tet(M)
3,983,668	C→T	Δ				100%	K331K (AAG→AAA)	tet(M) ←	tetracycline resistance ribosomal protection protein Tet(M)
3,983,694	G→A	Δ				100%	L323L (CTA→TTA)	tet(M) ←	tetracycline resistance ribosomal protection protein Tet(M)
3,983,728	C→T	Δ				100%	G311G (GGG→GGA)	tet(M) ←	tetracycline resistance ribosomal protection protein Tet(M)
3,983,731	G→A	Δ				100%	S310S (TCC→TCT)	tet(M) ←	tetracycline resistance ribosomal protection protein Tet(M)
3,983,740	2 bp→TC	Δ				100%	coding (920-921/1920 nt)	tet(M) ←	tetracycline resistance ribosomal protection protein Tet(M)
3,983,749	T→C	Δ				100%	K304K (AAA→AAG)	tet(M) ←	tetracycline resistance ribosomal protection protein Tet(M)
3,983,790	T→C	Δ				100%	I291V (ATT→GTT)	tet(M) ←	tetracycline resistance ribosomal protection protein Tet(M)
3,983,803	C→T	Δ				100%	K286K (AAG→AAA)	tet(M) ←	tetracycline resistance ribosomal protection protein Tet(M)
3,983,809	C→T	Δ				100%	S284S (TCG→TCA)	tet(M) ←	tetracycline resistance ribosomal protection protein Tet(M)
3,983,814	T→C	Δ				100%	I283V (ATA→GTA)	tet(M) ←	tetracycline resistance ribosomal protection protein Tet(M)
3,983,827	C→T	Δ				100%	R278R (CGG→CGA)	tet(M) ←	tetracycline resistance ribosomal protection protein Tet(M)
3,983,830	C→T	Δ				100%	L277L (TTG→TTA)	tet(M) ←	tetracycline resistance ribosomal protection protein Tet(M)
3,983,836	C→T	Δ				100%	L275L (CTG→CTA)	tet(M) ←	tetracycline resistance ribosomal protection protein Tet(M)
3,983,839	G→T	Δ				100%	I274V (ATC→GTA)	tet(M) ←	tetracycline resistance ribosomal protection protein Tet(M)
3,983,841	T→C	Δ				100%	I274V (ATC→GTA)	tet(M) ←	tetracycline resistance ribosomal protection protein Tet(M)
3,983,845	G→A	Δ				100%	G272S (GGC→AGT)	tet(M) ←	tetracycline resistance ribosomal protection protein Tet(M)
3,983,847	C→T	Δ				100%	G272S (GGC→AGT)	tet(M) ←	tetracycline resistance ribosomal protection protein Tet(M)
3,983,859	C→T	Δ				100%	V268I (GTA→ATA)	tet(M) ←	tetracycline resistance ribosomal protection protein Tet(M)
3,983,880	C→T	Δ				100%	E261K (GAA→AAA)	tet(M) ←	tetracycline resistance ribosomal protection protein Tet(M)
3,983,883	2 bp→TT	Δ				100%	coding (777-778/1920 nt)	tet(M) ←	tetracycline resistance ribosomal protection protein Tet(M)
3,983,886	A→T	Δ				100%	S259T (TCG→ACG)	tet(M) ←	tetracycline resistance ribosomal protection protein Tet(M)
3,983,902	G→A	Δ				100%	V253V (GTC→GTT)	tet(M) ←	tetracycline resistance ribosomal protection protein Tet(M)
3,983,906	A→T	Δ				100%	I252N (ATT→AAT)	tet(M) ←	tetracycline resistance ribosomal protection protein Tet(M)
3,983,924	5 bp→5 bp	Δ				100%	coding (733-737/1920 nt)	tet(M) ←	tetracycline resistance ribosomal protection protein Tet(M)
3,983,931	2 bp→GA	Δ				100%	coding (729-730/1920 nt)	tet(M) ←	tetracycline resistance ribosomal protection protein Tet(M)
3,983,934	A→G	Δ				100%	Y243H (TAC→CAC)	tet(M) ←	tetracycline resistance ribosomal protection protein Tet(M)
3,983,956	C→A	Δ				100%	T235T (ACG→ACT)	tet(M) ←	tetracycline resistance ribosomal protection protein Tet(M)
3,983,962	C→A	Δ				100%	V233V (GTG→GTT)	tet(M) ←	tetracycline resistance ribosomal protection protein Tet(M)
3,983,974	C→G	Δ				100%	Q229H (CAG→CAC)	tet(M) ←	tetracycline resistance ribosomal protection protein Tet(M)
3,983,976	2 bp→TA	Δ				100%	coding (684-685/1920 nt)	tet(M) ←	tetracycline resistance ribosomal protection protein Tet(M)
3,983,989	G→A	Δ				100%	N224N (AAC→AAT)	tet(M) ←	tetracycline resistance ribosomal protection protein Tet(M)
3,983,992	G→A	Δ				100%	S223S (AGC→AGT)	tet(M) ←	tetracycline resistance ribosomal protection protein Tet(M)
3,984,001	G→A	Δ				100%	S220S (AGC→AGT)	tet(M) ←	tetracycline resistance ribosomal protection protein Tet(M)
3,984,015	C→G	Δ				100%	V216L (GTT→CTT)	tet(M) ←	tetracycline resistance ribosomal protection protein Tet(M)
3,984,020	T→A	Δ				100%	Y214F (TAC→TTC)	tet(M) ←	tetracycline resistance ribosomal protection protein Tet(M)
3,984,024	2 bp→GA	Δ				100%	coding (636-637/1920 nt)	tet(M) ←	tetracycline resistance ribosomal protection protein Tet(M)
3,984,028	G→A	Δ				100%	C211C (TGC→TGT)	tet(M) ←	tetracycline resistance ribosomal protection protein Tet(M)
3,984,044	C→A	Δ				100%	R206I (AGA→ATA)	tet(M) ←	tetracycline resistance ribosomal protection protein Tet(M)
3,984,047	A→C	Δ				100%	I205S (ATC→AGC)	tet(M) ←	tetracycline resistance ribosomal protection protein Tet(M)
3,984,161	A→G	Δ				100%	M167T (ATG→ACG)	tet(M) ←	tetracycline resistance ribosomal protection protein Tet(M)
3,984,163	T→C	Δ				100%	V166V (GTA→GTG)	tet(M) ←	tetracycline resistance ribosomal protection protein Tet(M)
3,984,168	G→A	Δ				100%	R165C (CGT→TGT)	tet(M) ←	tetracycline resistance ribosomal protection protein Tet(M)
3,984,171	T→C	Δ				100%	M164V (ATG→GTG)	tet(M) ←	tetracycline resistance ribosomal protection protein Tet(M)
3,984,180	G→A	Δ				100%	H161Y (CAT→TAT)	tet(M) ←	tetracycline resistance ribosomal protection protein Tet(M)
3,984,211	C→G	Δ				100%	A150A (GCG→GCC)	tet(M) ←	tetracycline resistance ribosomal protection protein Tet(M)
3,984,682	A→G	Δ				100%	intergenic (-22/+226)	tet(M) ← / ← JMW53_18225	tetracycline resistance ribosomal protection protein Tet(M)/conjugal transfer protein
3,984,727	T→C	Δ				100%	intergenic (-67/+181)	tet(M) ← / ← JMW53_18225	tetracycline resistance ribosomal protection protein Tet(M)/conjugal transfer protein
3,984,742	A→G	Δ				100%	intergenic (-82/+166)	tet(M) ← / ← JMW53_18225	tetracycline resistance ribosomal protection protein Tet(M)/conjugal transfer protein
3,984,767	G→T	Δ				100%	intergenic (-107/+141)	tet(M) ← / ← JMW53_18225	tetracycline resistance ribosomal protection protein Tet(M)/conjugal transfer protein
3,984,801	T→A	Δ				100%	intergenic (-141/+107)	tet(M) ← / ← JMW53_18225	tetracycline resistance ribosomal protection protein Tet(M)/conjugal transfer protein
3,984,805	2 bp→GA	Δ				100%	intergenic (-145/+102)	tet(M) ← / ← JMW53_18225	tetracycline resistance ribosomal protection protein Tet(M)/conjugal transfer protein
3,984,809	2 bp→CT	Δ				100%	intergenic (-149/+98)	tet(M) ← / ← JMW53_18225	tetracycline resistance ribosomal protection protein Tet(M)/conjugal transfer protein
3,984,812	T→A	Δ				100%	intergenic (-152/+96)	tet(M) ← / ← JMW53_18225	tetracycline resistance ribosomal protection protein Tet(M)/conjugal transfer protein
3,984,815	C→T	Δ				100%	intergenic (-155/+93)	tet(M) ← / ← JMW53_18225	tetracycline resistance ribosomal protection protein Tet(M)/conjugal transfer protein
3,984,817	T→A	Δ				100%	intergenic (-157/+91)	tet(M) ← / ← JMW53_18225	tetracycline resistance ribosomal protection protein Tet(M)/conjugal transfer protein
3,984,824	A→G	Δ				100%	intergenic (-164/+84)	tet(M) ← / ← JMW53_18225	tetracycline resistance ribosomal protection protein Tet(M)/conjugal transfer protein
3,984,831	T→C	Δ				100%	intergenic (-171/+77)	tet(M) ← / ← JMW53_18225	tetracycline resistance ribosomal protection protein Tet(M)/conjugal transfer protein
3,984,844	2 bp→GA	Δ				100%	intergenic (-184/+63)	tet(M) ← / ← JMW53_18225	tetracycline resistance ribosomal protection protein Tet(M)/conjugal transfer protein
3,984,848	A→C	Δ				100%	intergenic (-188/+60)	tet(M) ← / ← JMW53_18225	tetracycline resistance ribosomal protection protein Tet(M)/conjugal transfer protein
3,984,852	G→A	Δ				100%	intergenic (-192/+56)	tet(M) ← / ← JMW53_18225	tetracycline resistance ribosomal protection protein Tet(M)/conjugal transfer protein
3,984,860	G→A	Δ				100%	intergenic (-200/+48)	tet(M) ← / ← JMW53_18225	tetracycline resistance ribosomal protection protein Tet(M)/conjugal transfer protein
3,984,866	+A	Δ				100%	intergenic (-206/+42)	tet(M) ← / ← JMW53_18225	tetracycline resistance ribosomal protection protein Tet(M)/conjugal transfer protein
3,984,870	A→T	Δ				100%	intergenic (-210/+38)	tet(M) ← / ← JMW53_18225	tetracycline resistance ribosomal protection protein Tet(M)/conjugal transfer protein
3,984,876	G→A	Δ				100%	intergenic (-216/+32)	tet(M) ← / ← JMW53_18225	tetracycline resistance ribosomal protection protein Tet(M)/conjugal transfer protein
3,984,890	A→G	Δ				100%	intergenic (-230/+18)	tet(M) ← / ← JMW53_18225	tetracycline resistance ribosomal protection protein Tet(M)/conjugal transfer protein
3,984,894	2 bp→TA	Δ				100%	intergenic (-234/+13)	tet(M) ← / ← JMW53_18225	tetracycline resistance ribosomal protection protein Tet(M)/conjugal transfer protein
3,984,902	T→C	Δ				100%	intergenic (-242/+6)	tet(M) ← / ← JMW53_18225	tetracycline resistance ribosomal protection protein Tet(M)/conjugal transfer protein
3,984,905	A→T	Δ				100%	intergenic (-245/+3)	tet(M) ← / ← JMW53_18225	tetracycline resistance ribosomal protection protein Tet(M)/conjugal transfer protein
3,984,906	+TTT	Δ				100%	intergenic (-246/+2)	tet(M) ← / ← JMW53_18225	tetracycline resistance ribosomal protection protein Tet(M)/conjugal transfer protein
3,984,913	T→C	Δ				100%	K310E (AAA→GAA)	JMW53_18225 ←	conjugal transfer protein
3,984,926	G→A	Δ				100%	N305N (AAC→AAT)	JMW53_18225 ←	conjugal transfer protein
3,984,930	T→C	Δ				100%	N304S (AAT→AGT)	JMW53_18225 ←	conjugal transfer protein
3,984,932	T→C	Δ				100%	G303G (GGA→GGG)	JMW53_18225 ←	conjugal transfer protein
3,984,934	+G	Δ				100%	coding (907/933 nt)	JMW53_18225 ←	conjugal transfer protein
3,984,936	A→T	Δ				100%	V302E (GTA→GAA)	JMW53_18225 ←	conjugal transfer protein
3,984,942	$(T)_{5\to4}$	Δ				100%	coding (899/933 nt)	JMW53_18225 ←	conjugal transfer protein
3,984,944	T→A	Δ				100%	L299L (CTA→CTT)	JMW53_18225 ←	conjugal transfer protein
3,984,947	C→T	Δ				100%	V298V (GTG→GTA)	JMW53_18225 ←	conjugal transfer protein
3,984,968	T→C	Δ				100%	Q291Q (CAA→CAG)	JMW53_18225 ←	conjugal transfer protein
3,984,994	G→T	Δ				100%	L283I (CTC→ATC)	JMW53_18225 ←	conjugal transfer protein
3,985,000	2 bp→CC	Δ				100%	coding (840-841/933 nt)	JMW53_18225 ←	conjugal transfer protein
3,985,010	T→C	Δ				100%	S277S (TCA→TCG)	JMW53_18225 ←	conjugal transfer protein
3,985,031	T→C	Δ				100%	K270K (AAA→AAG)	JMW53_18225 ←	conjugal transfer protein
3,985,042	2 bp→GA	Δ				100%	coding (798-799/933 nt)	JMW53_18225 ←	conjugal transfer protein
3,985,052	C→T	Δ				100%	V263V (GTG→GTA)	JMW53_18225 ←	conjugal transfer protein
3,985,055	G→C	Δ				100%	L262L (CTC→CTG)	JMW53_18225 ←	conjugal transfer protein
3,985,067	A→G	Δ				100%	I258I (ATT→ATC)	JMW53_18225 ←	conjugal transfer protein
3,985,070	A→G	Δ				100%	Y257Y (TAT→TAC)	JMW53_18225 ←	conjugal transfer protein
3,985,073	A→C	Δ				100%	D256E (GAT→GAG)	JMW53_18225 ←	conjugal transfer protein
3,985,085	A→T	Δ				100%	S252P (TCT→CCA)	JMW53_18225 ←	conjugal transfer protein
3,985,087	A→G	Δ				100%	S252P (TCT→CCA)	JMW53_18225 ←	conjugal transfer protein
3,985,091	C→T	Δ				100%	L250L (TTG→TTA)	JMW53_18225 ←	conjugal transfer protein
3,985,094	A→T	Δ				100%	I249I (ATT→ATA)	JMW53_18225 ←	conjugal transfer protein
3,985,097	A→C	Δ				100%	G248G (GGT→GGG)	JMW53_18225 ←	conjugal transfer protein
3,985,100	A→G	Δ				100%	D247D (GAT→GAC)	JMW53_18225 ←	conjugal transfer protein
3,985,115	A→G	Δ				100%	S242S (TCT→TCC)	JMW53_18225 ←	conjugal transfer protein
3,985,127	C→G	Δ				100%	A238A (GCG→GCC)	JMW53_18225 ←	conjugal transfer protein
3,985,133	T→C	Δ				100%	A236A (GCA→GCG)	JMW53_18225 ←	conjugal transfer protein
3,985,157	T→C	Δ				100%	T228T (ACA→ACG)	JMW53_18225 ←	conjugal transfer protein
3,985,165	G→A	Δ				100%	L226L (CTA→TTA)	JMW53_18225 ←	conjugal transfer protein
3,985,173	A→T	Δ				100%	I223N (ATT→AAT)	JMW53_18225 ←	conjugal transfer protein
3,985,184	T→G	Δ				100%	T219T (ACA→ACC)	JMW53_18225 ←	conjugal transfer protein
3,985,187	2 bp→TG	Δ				100%	coding (653-654/933 nt)	JMW53_18225 ←	conjugal transfer protein
3,985,202	T→C	Δ				100%	T213T (ACA→ACG)	JMW53_18225 ←	conjugal transfer protein
3,985,205	A→C	Δ				100%	G212G (GGT→GGG)	JMW53_18225 ←	conjugal transfer protein
3,985,208	A→C	Δ				100%	D211E (GAT→GAG)	JMW53_18225 ←	conjugal transfer protein
3,985,221	A→G	Δ				100%	V207A (GTC→GCC)	JMW53_18225 ←	conjugal transfer protein
3,985,235	3 bp→ACT	Δ				100%	coding (604-606/933 nt)	JMW53_18225 ←	conjugal transfer protein
3,985,254	C→T	Δ				100%	S196N (AGC→AAC)	JMW53_18225 ←	conjugal transfer protein
3,985,268	G→A	Δ				100%	N191N (AAC→AAT)	JMW53_18225 ←	conjugal transfer protein
3,985,271	T→C	Δ				100%	K190K (AAA→AAG)	JMW53_18225 ←	conjugal transfer protein
3,985,275	2 bp→AC	Δ				100%	coding (565-566/933 nt)	JMW53_18225 ←	conjugal transfer protein
3,985,310	2 bp→AC	Δ				100%	coding (530-531/933 nt)	JMW53_18225 ←	conjugal transfer protein
3,985,317	2 bp→AT	Δ				100%	coding (523-524/933 nt)	JMW53_18225 ←	conjugal transfer protein
3,985,328	A→G	Δ				100%	H171H (CAT→CAC)	JMW53_18225 ←	conjugal transfer protein
3,985,341	A→G	Δ				100%	I167T (ATA→ACA)	JMW53_18225 ←	conjugal transfer protein
3,985,349	C→T	Δ				100%	G164G (GGG→GGA)	JMW53_18225 ←	conjugal transfer protein
3,985,361	C→G	Δ				100%	L160L (TTG→CTC)	JMW53_18225 ←	conjugal transfer protein
3,985,363	A→G	Δ				100%	L160L (TTG→CTC)	JMW53_18225 ←	conjugal transfer protein
3,985,376	A→G	Δ				100%	Y155Y (TAT→TAC)	JMW53_18225 ←	conjugal transfer protein
3,985,382	C→T	Δ				100%	V153V (GTG→GTA)	JMW53_18225 ←	conjugal transfer protein
3,985,387	C→T	Δ				100%	D152N (GAT→AAT)	JMW53_18225 ←	conjugal transfer protein
3,985,393	2 bp→CA	Δ				100%	coding (447-448/933 nt)	JMW53_18225 ←	conjugal transfer protein
3,985,397	T→G	Δ				100%	E148D (GAA→GAC)	JMW53_18225 ←	conjugal transfer protein
3,985,404	2 bp→GT	Δ				100%	coding (436-437/933 nt)	JMW53_18225 ←	conjugal transfer protein
3,985,408	2 bp→GC	Δ				100%	coding (432-433/933 nt)	JMW53_18225 ←	conjugal transfer protein
3,985,411	T→C	Δ				100%	K144E (AAA→GAA)	JMW53_18225 ←	conjugal transfer protein
3,985,416	A→G	Δ				100%	M142T (ATG→ACG)	JMW53_18225 ←	conjugal transfer protein
3,985,429	2 bp→AT	Δ				100%	coding (411-412/933 nt)	JMW53_18225 ←	conjugal transfer protein
3,985,460	C→T	Δ				100%	K127K (AAG→AAA)	JMW53_18225 ←	conjugal transfer protein
3,985,463	T→G	Δ				100%	R126R (CGA→CGC)	JMW53_18225 ←	conjugal transfer protein
3,985,466	G→T	Δ				100%	V125V (GTC→GTA)	JMW53_18225 ←	conjugal transfer protein
3,985,477	T→C	Δ				100%	I122V (ATT→GTT)	JMW53_18225 ←	conjugal transfer protein
3,985,496	C→A	Δ				100%	E115D (GAG→GAT)	JMW53_18225 ←	conjugal transfer protein
3,985,509	G→C	Δ				100%	A111G (GCC→GGC)	JMW53_18225 ←	conjugal transfer protein
3,985,516	3 bp→GAC	Δ				100%	coding (323-325/933 nt)	JMW53_18225 ←	conjugal transfer protein
3,985,526	A→T	Δ				100%	R105R (CGT→CGA)	JMW53_18225 ←	conjugal transfer protein
3,985,543	2 bp→TA	Δ				100%	coding (297-298/933 nt)	JMW53_18225 ←	conjugal transfer protein
3,985,548	2 bp→GA	Δ				100%	coding (292-293/933 nt)	JMW53_18225 ←	conjugal transfer protein
3,985,551	A→T	Δ				100%	L97Q (CTA→CAA)	JMW53_18225 ←	conjugal transfer protein
3,985,553	T→C	Δ				100%	E96E (GAA→GAG)	JMW53_18225 ←	conjugal transfer protein
3,985,568	A→G	Δ				100%	V91V (GTT→GTC)	JMW53_18225 ←	conjugal transfer protein
3,985,603	T→G	Δ				100%	N80H (AAT→CAT)	JMW53_18225 ←	conjugal transfer protein
3,985,616	A→G	Δ				100%	Y75Y (TAT→TAC)	JMW53_18225 ←	conjugal transfer protein
3,985,627	T→C	Δ				100%	K72E (AAA→GAA)	JMW53_18225 ←	conjugal transfer protein
3,985,634	G→A	Δ				100%	T69T (ACC→ACT)	JMW53_18225 ←	conjugal transfer protein
3,985,649	G→A	Δ				100%	T64T (ACC→ACT)	JMW53_18225 ←	conjugal transfer protein
3,985,652	G→A	Δ				100%	H63H (CAC→CAT)	JMW53_18225 ←	conjugal transfer protein
3,985,655	G→A	Δ				100%	T62T (ACC→ACT)	JMW53_18225 ←	conjugal transfer protein
3,985,661	A→T	Δ				100%	V60V (GTT→GTA)	JMW53_18225 ←	conjugal transfer protein
3,985,663	2 bp→TA	Δ				100%	coding (177-178/933 nt)	JMW53_18225 ←	conjugal transfer protein
3,985,679	A→G	Δ				100%	F54F (TTT→TTC)	JMW53_18225 ←	conjugal transfer protein
3,985,685	T→C	Δ				100%	A52A (GCA→GCG)	JMW53_18225 ←	conjugal transfer protein
3,985,712	C→T	Δ				100%	V43V (GTG→GTA)	JMW53_18225 ←	conjugal transfer protein
3,985,718	T→C	Δ				100%	L41L (TTA→TTG)	JMW53_18225 ←	conjugal transfer protein
3,985,722	C→G	Δ				100%	G40A (GGC→GCC)	JMW53_18225 ←	conjugal transfer protein
3,985,740	C→T	Δ				100%	R34H (CGT→CAT)	JMW53_18225 ←	conjugal transfer protein
3,985,751	T→C	Δ				100%	K30K (AAA→AAG)	JMW53_18225 ←	conjugal transfer protein
3,985,754	G→A	Δ				100%	Y29Y (TAC→TAT)	JMW53_18225 ←	conjugal transfer protein
3,985,759	T→C	Δ				100%	I28V (ATC→GTC)	JMW53_18225 ←	conjugal transfer protein
3,985,811	G→A	Δ				100%	F10F (TTC→TTT)	JMW53_18225 ←	conjugal transfer protein
3,985,824	G→A	Δ				100%	S6L (TCA→TTA)	JMW53_18225 ←	conjugal transfer protein
3,985,861	T→C	Δ				100%	V326V (GTA→GTG)	JMW53_18230 ←	bifunctional lysozyme/C40 family peptidase
3,985,879	A→G	Δ				100%	Y320Y (TAT→TAC)	JMW53_18230 ←	bifunctional lysozyme/C40 family peptidase
3,985,884	A→G	Δ				100%	S319P (TCC→CCC)	JMW53_18230 ←	bifunctional lysozyme/C40 family peptidase
3,985,903	G→A	Δ				100%	G312G (GGC→GGT)	JMW53_18230 ←	bifunctional lysozyme/C40 family peptidase
3,985,909	A→T	Δ				100%	P310P (CCT→CCA)	JMW53_18230 ←	bifunctional lysozyme/C40 family peptidase
3,985,915	T→G	Δ				100%	G308G (GGA→GGC)	JMW53_18230 ←	bifunctional lysozyme/C40 family peptidase
3,985,921	G→A	Δ				100%	H306H (CAC→CAT)	JMW53_18230 ←	bifunctional lysozyme/C40 family peptidase
3,985,930	T→A	Δ				100%	R303R (CGA→CGT)	JMW53_18230 ←	bifunctional lysozyme/C40 family peptidase
3,985,933	A→G	Δ				100%	N302N (AAT→AAC)	JMW53_18230 ←	bifunctional lysozyme/C40 family peptidase
3,985,938	C→T	Δ				100%	D301N (GAT→AAT)	JMW53_18230 ←	bifunctional lysozyme/C40 family peptidase
3,985,942	T→A	Δ				100%	L299F (TTA→TTT)	JMW53_18230 ←	bifunctional lysozyme/C40 family peptidase
3,985,944	2 bp→GG	Δ				100%	coding (894-895/1002 nt)	JMW53_18230 ←	bifunctional lysozyme/C40 family peptidase
3,985,948	A→T	Δ				100%	I297I (ATT→ATA)	JMW53_18230 ←	bifunctional lysozyme/C40 family peptidase
3,985,954	G→A	Δ				100%	I295V (ATC→GTT)	JMW53_18230 ←	bifunctional lysozyme/C40 family peptidase
3,985,956	T→C	Δ				100%	I295V (ATC→GTT)	JMW53_18230 ←	bifunctional lysozyme/C40 family peptidase
3,986,016	T→G	Δ				100%	K275Q (AAA→CAA)	JMW53_18230 ←	bifunctional lysozyme/C40 family peptidase
3,986,023	G→C	Δ				100%	S272S (TCC→TCG)	JMW53_18230 ←	bifunctional lysozyme/C40 family peptidase
3,986,038	T→C	Δ				100%	Q267Q (CAA→CAG)	JMW53_18230 ←	bifunctional lysozyme/C40 family peptidase
3,986,056	T→C	Δ				100%	Q261Q (CAA→CAG)	JMW53_18230 ←	bifunctional lysozyme/C40 family peptidase
3,986,077	T→G	Δ				100%	K254N (AAA→AAC)	JMW53_18230 ←	bifunctional lysozyme/C40 family peptidase
3,986,083	C→T	Δ				100%	G252G (GGG→GGA)	JMW53_18230 ←	bifunctional lysozyme/C40 family peptidase
3,986,092	G→T	Δ				100%	G249G (GGC→GGA)	JMW53_18230 ←	bifunctional lysozyme/C40 family peptidase
3,986,128	T→A	Δ				100%	S237S (TCA→TCT)	JMW53_18230 ←	bifunctional lysozyme/C40 family peptidase
3,986,149	G→A	Δ				100%	G230G (GGC→GGT)	JMW53_18230 ←	bifunctional lysozyme/C40 family peptidase
3,986,152	A→G	Δ				100%	Y229Y (TAT→TAC)	JMW53_18230 ←	bifunctional lysozyme/C40 family peptidase
3,986,155	G→A	Δ				100%	V228V (GTC→GTT)	JMW53_18230 ←	bifunctional lysozyme/C40 family peptidase
3,986,158	A→G	Δ				100%	Y227Y (TAT→TAC)	JMW53_18230 ←	bifunctional lysozyme/C40 family peptidase
3,986,161	3 bp→TCG	Δ				100%	coding (676-678/1002 nt)	JMW53_18230 ←	bifunctional lysozyme/C40 family peptidase
3,986,167	A→G	Δ				100%	G224G (GGT→GGC)	JMW53_18230 ←	bifunctional lysozyme/C40 family peptidase
3,986,211	T→C	Δ				100%	N210D (AAT→GAT)	JMW53_18230 ←	bifunctional lysozyme/C40 family peptidase
3,986,217	T→C	Δ				100%	N208D (AAT→GAT)	JMW53_18230 ←	bifunctional lysozyme/C40 family peptidase
3,986,221	T→C	Δ				100%	Q206Q (CAA→CAG)	JMW53_18230 ←	bifunctional lysozyme/C40 family peptidase
3,986,223	2 bp→CT	Δ				100%	coding (615-616/1002 nt)	JMW53_18230 ←	bifunctional lysozyme/C40 family peptidase
3,986,245	A→T	Δ				100%	V198V (GTT→GTA)	JMW53_18230 ←	bifunctional lysozyme/C40 family peptidase
3,986,248	A→C	Δ				100%	L197L (CTT→CTG)	JMW53_18230 ←	bifunctional lysozyme/C40 family peptidase
3,986,251	C→T	Δ				100%	Q196Q (CAG→CAA)	JMW53_18230 ←	bifunctional lysozyme/C40 family peptidase
3,986,254	A→C	Δ				100%	V195V (GTT→GTG)	JMW53_18230 ←	bifunctional lysozyme/C40 family peptidase
3,986,278	A→G	Δ				100%	Y187Y (TAT→TAC)	JMW53_18230 ←	bifunctional lysozyme/C40 family peptidase
3,986,299	G→A	Δ				100%	P180P (CCC→CCT)	JMW53_18230 ←	bifunctional lysozyme/C40 family peptidase
3,986,311	T→G	Δ				100%	P176P (CCA→CCC)	JMW53_18230 ←	bifunctional lysozyme/C40 family peptidase
3,986,320	A→G	Δ				100%	Y173Y (TAT→TAC)	JMW53_18230 ←	bifunctional lysozyme/C40 family peptidase
3,986,323	G→A	Δ				100%	S172S (TCC→TCT)	JMW53_18230 ←	bifunctional lysozyme/C40 family peptidase
3,986,338	2 bp→AC	Δ				100%	coding (500-501/1002 nt)	JMW53_18230 ←	bifunctional lysozyme/C40 family peptidase
3,986,347	T→C	Δ				100%	E164E (GAA→GAG)	JMW53_18230 ←	bifunctional lysozyme/C40 family peptidase
3,986,350	C→T	Δ				100%	K163K (AAG→AAA)	JMW53_18230 ←	bifunctional lysozyme/C40 family peptidase
3,986,353	A→T	Δ				100%	S162S (TCT→TCA)	JMW53_18230 ←	bifunctional lysozyme/C40 family peptidase
3,986,386	G→A	Δ				100%	N151N (AAC→AAT)	JMW53_18230 ←	bifunctional lysozyme/C40 family peptidase
3,986,416	G→A	Δ				100%	G141G (GGC→GGT)	JMW53_18230 ←	bifunctional lysozyme/C40 family peptidase
3,986,422	G→A	Δ				100%	G139G (GGC→GGT)	JMW53_18230 ←	bifunctional lysozyme/C40 family peptidase
3,986,440	A→G	Δ				100%	I133I (ATT→ATC)	JMW53_18230 ←	bifunctional lysozyme/C40 family peptidase
3,986,452	C→T	Δ				100%	L129L (TTG→TTA)	JMW53_18230 ←	bifunctional lysozyme/C40 family peptidase
3,986,458	C→T	Δ				100%	V127V (GTG→GTA)	JMW53_18230 ←	bifunctional lysozyme/C40 family peptidase
3,986,463	C→T	Δ				100%	G126S (GGT→AGT)	JMW53_18230 ←	bifunctional lysozyme/C40 family peptidase
3,986,470	A→T	Δ				100%	D123E (GAT→GAA)	JMW53_18230 ←	bifunctional lysozyme/C40 family peptidase
3,986,473	T→G	Δ				100%	R122S (AGA→AGC)	JMW53_18230 ←	bifunctional lysozyme/C40 family peptidase
3,986,515	G→A	Δ				100%	I108I (ATC→ATT)	JMW53_18230 ←	bifunctional lysozyme/C40 family peptidase
3,986,518	A→G	Δ				100%	S107S (TCT→TCC)	JMW53_18230 ←	bifunctional lysozyme/C40 family peptidase
3,986,542	G→A	Δ				100%	P99P (CCC→CCT)	JMW53_18230 ←	bifunctional lysozyme/C40 family peptidase
3,986,548	T→A	Δ				100%	L97L (CTA→CTT)	JMW53_18230 ←	bifunctional lysozyme/C40 family peptidase
3,986,554	A→G	Δ				100%	L95L (CTT→CTC)	JMW53_18230 ←	bifunctional lysozyme/C40 family peptidase
3,986,584	G→C	Δ				100%	A85A (GCC→GCG)	JMW53_18230 ←	bifunctional lysozyme/C40 family peptidase
3,986,614	A→C	Δ				100%	A75A (GCT→GCG)	JMW53_18230 ←	bifunctional lysozyme/C40 family peptidase
3,986,620	C→A	Δ				100%	L73L (CTG→CTT)	JMW53_18230 ←	bifunctional lysozyme/C40 family peptidase
3,986,625	2 bp→TG	Δ				100%	coding (213-214/1002 nt)	JMW53_18230 ←	bifunctional lysozyme/C40 family peptidase
3,986,629	A→G	Δ				100%	V70V (GTT→GTC)	JMW53_18230 ←	bifunctional lysozyme/C40 family peptidase
3,986,632	G→A	Δ				100%	Y69Y (TAC→TAT)	JMW53_18230 ←	bifunctional lysozyme/C40 family peptidase
3,986,643	T→C	Δ				100%	I66V (ATT→GTT)	JMW53_18230 ←	bifunctional lysozyme/C40 family peptidase
3,986,654	C→T	Δ				100%	R62K (AGA→AAA)	JMW53_18230 ←	bifunctional lysozyme/C40 family peptidase
3,986,656	A→G	Δ				100%	A61A (GCT→GCC)	JMW53_18230 ←	bifunctional lysozyme/C40 family peptidase
3,986,659	G→A	Δ				100%	Y60Y (TAC→TAT)	JMW53_18230 ←	bifunctional lysozyme/C40 family peptidase
3,986,713	G→T	Δ				100%	G42G (GGC→GGA)	JMW53_18230 ←	bifunctional lysozyme/C40 family peptidase
3,986,719	G→A	Δ				100%	H40H (CAC→CAT)	JMW53_18230 ←	bifunctional lysozyme/C40 family peptidase
3,986,725	G→A	Δ				100%	N38N (AAC→AAT)	JMW53_18230 ←	bifunctional lysozyme/C40 family peptidase
3,986,728	T→G	Δ				100%	S37S (TCA→TCC)	JMW53_18230 ←	bifunctional lysozyme/C40 family peptidase
3,986,733	2 bp→TT	Δ				100%	coding (105-106/1002 nt)	JMW53_18230 ←	bifunctional lysozyme/C40 family peptidase
3,986,739	2 bp→TG	Δ				100%	coding (99-100/1002 nt)	JMW53_18230 ←	bifunctional lysozyme/C40 family peptidase
3,986,743	T→C	Δ				100%	Q32Q (CAA→CAG)	JMW53_18230 ←	bifunctional lysozyme/C40 family peptidase
3,986,779	T→C	Δ				100%	L20L (CTA→CTG)	JMW53_18230 ←	bifunctional lysozyme/C40 family peptidase
3,986,802	2 bp→AT	Δ				100%	coding (36-37/1002 nt)	JMW53_18230 ←	bifunctional lysozyme/C40 family peptidase
3,986,826	C→T	Δ				100%	A5T (GCT→ACT)	JMW53_18230 ←	bifunctional lysozyme/C40 family peptidase
3,986,832	2 bp→AC	Δ				100%	coding (6-7/1002 nt)	JMW53_18230 ←	bifunctional lysozyme/C40 family peptidase
3,986,849	T→C	Δ				100%	S722G (AGT→GGT)	JMW53_18235 ←	YtxH domain-containing protein
3,986,851	C→A	Δ				100%	G721V (GGC→GTC)	JMW53_18235 ←	YtxH domain-containing protein
3,986,859	G→A	Δ				100%	I718I (ATC→ATT)	JMW53_18235 ←	YtxH domain-containing protein
3,986,862	A→G	Δ				100%	T717T (ACT→ACC)	JMW53_18235 ←	YtxH domain-containing protein
3,986,870	A→T	Δ				100%	S715T (TCC→ACC)	JMW53_18235 ←	YtxH domain-containing protein
3,986,874	T→G	Δ				100%	I713I (ATA→ATC)	JMW53_18235 ←	YtxH domain-containing protein
3,986,887	T→C	Δ				100%	N709S (AAT→AGT)	JMW53_18235 ←	YtxH domain-containing protein
3,986,892	4 bp→TGTA	Δ				100%	coding (2118-2121/2178 nt)	JMW53_18235 ←	YtxH domain-containing protein
3,986,901	3 bp→TAG	Δ				100%	coding (2110-2112/2178 nt)	JMW53_18235 ←	YtxH domain-containing protein
3,986,905	T→G	Δ				100%	Q703P (CAA→CCA)	JMW53_18235 ←	YtxH domain-containing protein
3,986,907	A→T	Δ				100%	V702V (GTT→GTA)	JMW53_18235 ←	YtxH domain-containing protein
3,986,931	C→T	Δ				100%	E694E (GAG→GAA)	JMW53_18235 ←	YtxH domain-containing protein
3,986,942	C→T	Δ				100%	V691I (GTC→ATC)	JMW53_18235 ←	YtxH domain-containing protein
3,986,957	C→T	Δ				100%	A686T (GCT→ACT)	JMW53_18235 ←	YtxH domain-containing protein
3,986,961	G→A	Δ				100%	D684D (GAC→GAT)	JMW53_18235 ←	YtxH domain-containing protein
3,986,969	2 bp→TA	Δ				100%	coding (2043-2044/2178 nt)	JMW53_18235 ←	YtxH domain-containing protein
3,986,973	A→G	Δ				100%	T680T (ACT→ACC)	JMW53_18235 ←	YtxH domain-containing protein
3,986,997	T→G	Δ				100%	T672T (ACA→ACC)	JMW53_18235 ←	YtxH domain-containing protein
3,987,001	C→T	Δ				100%	R671K (AGA→AAA)	JMW53_18235 ←	YtxH domain-containing protein
3,987,009	4 bp→AACG	Δ				100%	coding (2001-2004/2178 nt)	JMW53_18235 ←	YtxH domain-containing protein
3,987,016	2 bp→TT	Δ				100%	coding (1996-1997/2178 nt)	JMW53_18235 ←	YtxH domain-containing protein
3,987,019	2 bp→CC	Δ				100%	coding (1993-1994/2178 nt)	JMW53_18235 ←	YtxH domain-containing protein
3,987,024	T→G	Δ				100%	R663R (CGA→CGC)	JMW53_18235 ←	YtxH domain-containing protein
3,987,031	G→A	Δ				100%	A661V (GCG→GTG)	JMW53_18235 ←	YtxH domain-containing protein
3,987,037	2 bp→GG	Δ				100%	coding (1975-1976/2178 nt)	JMW53_18235 ←	YtxH domain-containing protein
3,987,043	A→G	Δ				100%	L657S (TTA→TCA)	JMW53_18235 ←	YtxH domain-containing protein
3,987,046	G→A	Δ				100%	S656F (TCT→TTT)	JMW53_18235 ←	YtxH domain-containing protein
3,987,072	C→T	Δ				100%	Q647Q (CAG→CAA)	JMW53_18235 ←	YtxH domain-containing protein
3,987,094	A→T	Δ				100%	M640K (ATG→AAG)	JMW53_18235 ←	YtxH domain-containing protein
3,987,096	3 bp→ATA	Δ				100%	coding (1915-1917/2178 nt)	JMW53_18235 ←	YtxH domain-containing protein
3,987,112	2 bp→TT	Δ				100%	coding (1900-1901/2178 nt)	JMW53_18235 ←	YtxH domain-containing protein
3,987,117	T→C	Δ				100%	Q632Q (CAA→CAG)	JMW53_18235 ←	YtxH domain-containing protein
3,987,125	C→A	Δ				100%	A630S (GCA→TCA)	JMW53_18235 ←	YtxH domain-containing protein
3,987,133	C→T	Δ				100%	G627E (GGA→GAA)	JMW53_18235 ←	YtxH domain-containing protein
3,987,142	G→T	Δ				100%	S624Y (TCT→TAT)	JMW53_18235 ←	YtxH domain-containing protein
3,987,152	+T	Δ				100%	coding (1861/2178 nt)	JMW53_18235 ←	YtxH domain-containing protein
3,987,156	Δ1 bp	Δ				100%	coding (1857/2178 nt)	JMW53_18235 ←	YtxH domain-containing protein
3,987,161	2 bp→TC	Δ				100%	coding (1851-1852/2178 nt)	JMW53_18235 ←	YtxH domain-containing protein
3,987,177	G→A	Δ				100%	S612S (TCC→TCT)	JMW53_18235 ←	YtxH domain-containing protein
3,987,186	T→A	Δ				100%	P609P (CCA→CCT)	JMW53_18235 ←	YtxH domain-containing protein
3,987,230	2 bp→TC	Δ				100%	coding (1782-1783/2178 nt)	JMW53_18235 ←	YtxH domain-containing protein
3,987,243	G→T	Δ				100%	R590R (CGC→CGA)	JMW53_18235 ←	YtxH domain-containing protein
3,987,246	T→C	Δ				100%	R589R (AGA→AGG)	JMW53_18235 ←	YtxH domain-containing protein
3,987,273	G→A	Δ				100%	S580S (AGC→AGT)	JMW53_18235 ←	YtxH domain-containing protein
3,987,285	T→C	Δ				100%	A576A (GCA→GCG)	JMW53_18235 ←	YtxH domain-containing protein
3,987,289	C→T	Δ				100%	R575K (AGA→AAA)	JMW53_18235 ←	YtxH domain-containing protein
3,987,306	4 bp→ACTG	Δ				100%	coding (1704-1707/2178 nt)	JMW53_18235 ←	YtxH domain-containing protein
3,987,314	2 bp→AA	Δ				100%	coding (1698-1699/2178 nt)	JMW53_18235 ←	YtxH domain-containing protein
3,987,321	A→G	Δ				100%	V564V (GTT→GTC)	JMW53_18235 ←	YtxH domain-containing protein
3,987,327	T→C	Δ				100%	E562E (GAA→GAG)	JMW53_18235 ←	YtxH domain-containing protein
3,987,334	G→A	Δ				100%	A560V (GCA→GTA)	JMW53_18235 ←	YtxH domain-containing protein
3,987,354	C→T	Δ				100%	V553V (GTG→GTA)	JMW53_18235 ←	YtxH domain-containing protein
3,987,378	A→C	Δ				100%	I545M (ATT→TTG)	JMW53_18235 ←	YtxH domain-containing protein
3,987,380	T→A	Δ				100%	I545M (ATT→TTG)	JMW53_18235 ←	YtxH domain-containing protein
3,987,384	2 bp→TG	Δ				100%	coding (1628-1629/2178 nt)	JMW53_18235 ←	YtxH domain-containing protein
3,987,399	G→C	Δ				100%	L538M (CTC→TTG)	JMW53_18235 ←	YtxH domain-containing protein
3,987,401	G→A	Δ				100%	L538M (CTC→TTG)	JMW53_18235 ←	YtxH domain-containing protein
3,987,403	2 bp→CC	Δ				100%	coding (1609-1610/2178 nt)	JMW53_18235 ←	YtxH domain-containing protein
3,987,407	C→T	Δ				100%	G536S (GGT→AGT)	JMW53_18235 ←	YtxH domain-containing protein
3,987,413	2 bp→TG	Δ				100%	coding (1599-1600/2178 nt)	JMW53_18235 ←	YtxH domain-containing protein
3,987,417	A→T	Δ				100%	V532V (GTT→GTA)	JMW53_18235 ←	YtxH domain-containing protein
3,987,420	G→C	Δ				100%	I531M (ATC→ATG)	JMW53_18235 ←	YtxH domain-containing protein
3,987,424	T→C	Δ				100%	K530R (AAA→AGA)	JMW53_18235 ←	YtxH domain-containing protein

3,987,426	A→G	Δ				100%	D529D (GAT→GAC)	*JMW53_18235* ←	YtxH domain-containing protein
3,987,441	A→T	Δ				100%	V524V (GTT→GTA)	*JMW53_18235* ←	YtxH domain-containing protein
3,987,446	2 bp→TA	Δ				100%	coding (1566-1567/2178 nt)	*JMW53_18235* ←	YtxH domain-containing protein
3,987,453	T→G	Δ				100%	T520T (ACA→ACC)	*JMW53_18235* ←	YtxH domain-containing protein
3,987,459	T→A	Δ				100%	G518G (GGA→GGT)	*JMW53_18235* ←	YtxH domain-containing protein
3,987,491	G→T	Δ				100%	Q508K (CAG→AAG)	*JMW53_18235* ←	YtxH domain-containing protein
3,987,501	A→T	Δ				100%	R504R (CGT→CGA)	*JMW53_18235* ←	YtxH domain-containing protein
3,987,506	T→A	Δ				100%	T503S (ACC→TCC)	*JMW53_18235* ←	YtxH domain-containing protein
3,987,576	G→C	Δ				100%	G479G (GGC→GGG)	*JMW53_18235* ←	YtxH domain-containing protein
3,987,582	A→C	Δ				100%	G477G (GGT→GGG)	*JMW53_18235* ←	YtxH domain-containing protein
3,987,587	A→G	Δ				100%	L476L (TTA→CTA)	*JMW53_18235* ←	YtxH domain-containing protein
3,987,590	2 bp→TA	Δ				100%	coding (1422-1423/2178 nt)	*JMW53_18235* ←	YtxH domain-containing protein
3,987,600	G→A	Δ				100%	R471R (CGC→CGT)	*JMW53_18235* ←	YtxH domain-containing protein
3,987,612	A→C	Δ				100%	R467R (CGT→CGG)	*JMW53_18235* ←	YtxH domain-containing protein
3,987,621	G→A	Δ				100%	R464R (CGC→CGT)	*JMW53_18235* ←	YtxH domain-containing protein
3,987,630	A→G	Δ				100%	H461H (CAT→CAC)	*JMW53_18235* ←	YtxH domain-containing protein
3,987,693	G→A	Δ				100%	S440S (AGC→AGT)	*JMW53_18235* ←	YtxH domain-containing protein
3,987,696	T→C	Δ				100%	Q439Q (CAA→CAG)	*JMW53_18235* ←	YtxH domain-containing protein
3,987,699	A→T	Δ				100%	L438L (CTT→CTA)	*JMW53_18235* ←	YtxH domain-containing protein
3,987,702	C→A	Δ				100%	S437S (TCG→TCT)	*JMW53_18235* ←	YtxH domain-containing protein
3,987,725	T→C	Δ				100%	S430G (AGC→GGC)	*JMW53_18235* ←	YtxH domain-containing protein
3,987,729	T→C	Δ				100%	K428K (AAA→AAG)	*JMW53_18235* ←	YtxH domain-containing protein
3,987,764	A→G	Δ				100%	L417L (TTA→CTA)	*JMW53_18235* ←	YtxH domain-containing protein
3,987,768	G→A	Δ				100%	A415A (GCC→GCT)	*JMW53_18235* ←	YtxH domain-containing protein
3,987,828	G→T	Δ				100%	A395A (GCC→GCA)	*JMW53_18235* ←	YtxH domain-containing protein
3,987,845	2 bp→AT	Δ				100%	coding (1167-1168/2178 nt)	*JMW53_18235* ←	YtxH domain-containing protein
4,003,188	A→G	100%					N201D (AAT→GAT)	*JMW53_18320* →	MFS transporter
4,021,765	A→G		100%				Y108C (TAC→TGC)	*JMW53_18405* →	GNAT family N-acetyltransferase
4,044,952	A→C	100%					intergenic (-76/+264)	*JMW53_18515* ← / ← *JMW53_18520*	lactate utilization protein/DUF4003 domain-containing protein
4,048,209	A→C	100%					I19S (ATT→AGT)	*JMW53_18535* ←	BlaI/MecI/CopY family transcriptional regulator
